# Pxmp2 Is a Channel-Forming Protein in Mammalian Peroxisomal Membrane

**DOI:** 10.1371/journal.pone.0005090

**Published:** 2009-04-07

**Authors:** Aare Rokka, Vasily D. Antonenkov, Raija Soininen, Hanna L. Immonen, Päivi L. Pirilä, Ulrich Bergmann, Raija T. Sormunen, Matti Weckström, Roland Benz, J. Kalervo Hiltunen

**Affiliations:** 1 Department of Biochemistry, Biocenter Oulu, University of Oulu, Oulu, Finland; 2 Department of Medical Biochemistry and Molecular Biology, Biocenter Oulu, University of Oulu, Oulu, Finland; 3 Department of Pathology, Biocenter Oulu, University of Oulu, Oulu, Finland; 4 Department of Physical Sciences, Biocenter Oulu, University of Oulu, Oulu, Finland; 5 Lehrstuhl fur Biotechnologie, Theodor-Boveri-Institut (Biozentrum) der Universität Wurzburg, Am Hubland, Wurzburg, Germany; Auburn University, United States of America

## Abstract

**Background:**

Peroxisomal metabolic machinery requires a continuous flow of organic and inorganic solutes across peroxisomal membrane. Concerning small solutes, the molecular nature of their traffic has remained an enigma.

**Methods/Principal Findings:**

In this study, we show that disruption in mice of the *Pxmp2* gene encoding Pxmp2, which belongs to a family of integral membrane proteins with unknown function, leads to partial restriction of peroxisomal membrane permeability to solutes *in vitro* and *in vivo*. Multiple-channel recording of liver peroxisomal preparations reveals that the channel-forming components with a conductance of 1.3 nS in 1.0 M KCl were lost in *Pxmp2* ^−/−^ mice. The channel-forming properties of Pxmp2 were confirmed with recombinant protein expressed in insect cells and with native Pxmp2 purified from mouse liver. The Pxmp2 channel, with an estimated diameter of 1.4 nm, shows weak cation selectivity and no voltage dependence. The long-lasting open states of the channel indicate its functional role as a protein forming a general diffusion pore in the membrane.

**Conclusions/Significance:**

Pxmp2 is the first peroxisomal channel identified, and its existence leads to prediction that the mammalian peroxisomal membrane is permeable to small solutes while transfer of “bulky” metabolites, e.g., cofactors (NAD/H, NADP/H, and CoA) and ATP, requires specific transporters.

## Introduction

Peroxisomes are small oxidative organelles found in all eukaryotes. They contain a matrix which is surrounded by a single membrane and consists mainly of soluble proteins. Peroxisomal enzymes are involved in a broad spectrum of metabolic pathways including conversion of lipids, amino- and hydroxyacids, purines and reactive oxygen species [1]. The carbon flow through peroxisomal pathways presupposes a continuous metabolite transfer across the peroxisomal membrane. The mechanism of this transfer represents a long-standing problem in the biology of peroxisomes. Two contrasting models have been discussed for more than 40 years: (1) the membrane of the peroxisome contains pore-forming proteins and is freely permeable to solutes, and (2) the peroxisomal membrane is completely impermeable to small solutes and contains a set of selective transporters (for review see [2], [3]).

Transporters specific for ATP have been characterized in yeast and mammalian peroxisomes [3], [4]. These findings were regarded as a proof that the membrane of these organelles is impermeable to solutes. However, other studies have shown that rat liver peroxisomal membrane forms a permeability barrier for cofactors and other ‘bulky’ solutes comparable by size with ATP but open for small metabolites *in vitro* [5]. An analysis of channel-forming activities in highly purified preparations of mouse liver peroxisomes suggested the presence of at least two types of channels allowing passage of solutes across the membrane [6]. These data are in line with findings indicating presence of the channel-forming proteins in plant peroxisomes [7], [8]. Likewise, the contradictory results were obtained when attempts have been made to establish an existence of pH [9], [10] or Ca^+2^ [11], [12] gradients across the peroxisomal membrane (see ‘Discussion’ for details).

Pxmp2, an integral membrane protein of peroxisomes with a monomeric molecular mass of 22 kDa, is abundant in rat liver [13], but less abundant in mouse liver [14]. Murine Pxmp2 consists of 194 amino acid residues with four putative transmembrane segments. Two additional Pxmp2 family members were identified in mammalian cells: the *Mpv17* gene product [15] and the Mpv17-like protein (M-LP, [16]). Initially, both proteins were localized to peroxisomal membrane [15], [16]. However, the localization of Mpv17 to peroxisomes has recently been challenged since the mammalian protein [17] and its yeast homolog Sym1p [18] were detectable in the inner mitochondrial membrane.

Pxmp2 was speculated previously as to having a role in the transmembrane transport of solutes by acting as a nonselective pore-forming protein [19]. This assumption was based on experiments showing that a protein fraction containing Pxmp2, PMP28, and some other peroxisomal membrane proteins from rat liver was able to promote leakage of small molecules such as sucrose from liposomes preloaded with these solutes. However, protein data-based analysis revealed that the Pxmp2 family members share no sequence or structural similarities with known porin proteins or other channels. Moreover, the presence of any type of pore-forming proteins in mammalian and yeast peroxisomes and their participation in the transfer of solutes across the membrane is widely challenged (for review see [2], [3]).

The present work addresses the molecular mechanism of transferring solutes across the peroxisomal membrane and the physiological role of the peroxisomal membrane protein Pxmp2. The data revealed that Pxmp2 is a channel-forming protein that functions as a size-selective filter with an exclusion limit of approximately 0.6 kDa for hydrophilic solutes.

## Results

### Pxmp2-deficient mice

**Figure 1:**
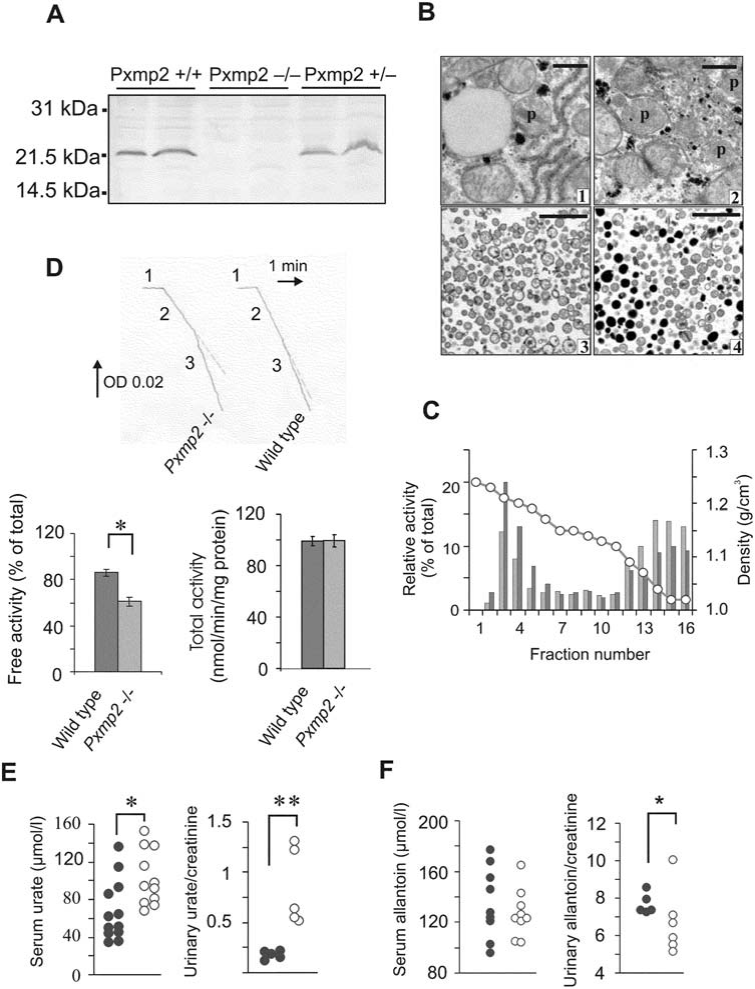
Characterization of Pxmp2 deficient peroxisomes and detection of metabolites in blood and urine. (A) Western blot analysis of postnuclear homogenates from livers of Pxmp2^+/+^, Pxmp2^−/−^, and Pxmp2^+/−^ mice, respectively, by using antibodies against Pxmp2. The molecular mass markers are indicated on the left. (B) Morphological examination of peroxisomes. (1) Peroxisome visible in liver cross section of wild-type mouse, p = peroxisome; (2) cluster of Pxmp2-deficient peroxisomes; (3,4) peroxisomes from livers of *Pxmp2* ^−/−^ (3) and wild-type (4) mice isolated using a Nycodenz gradient (see Figure 2B; fractions 3–4 were collected for analysis). Bar: 500 nm (1,2); 1000 nm (3,4). (C) Effect of Pxmp2 deletion on the integrity of peroxisomes *in vitro*. The postnuclear homogenates of liver samples were centrifuged using Nycodenz gradients. The activity of L-α-hydroxyacid oxidase was determined as a marker for soluble peroxisomal matrix proteins in both wild-type (black bars) and Pxmp2-deficient (gray bars) preparations. The ordinate axis (left) represents relative enzyme activities in each fraction (percentage of the whole activity loaded on the gradient which consisted of 0.22 units and 0.23 units for wild-type and Pxmp2-deficient samples, respectively). Recoveries of the enzyme were 106% (control) and 94% (*Pxmp2^−/−^*), respectively. The line connecting empty cycles indicates density of the gradient (shown on the right ordinate axis). Note an excessive leakage of L-α-hydroxyacid oxidase from Pxmp2-deficient peroxisomes. (D) Effect of Pxmp2 deletion on the latency of peroxisomal urate oxidase. Upper panel: absorbance traces at 292 nm indicating oxidation of uric acid. Incubation medium contained: purified peroxisomes (40 µg) only (1); + uric acid (2); + 0.05% (w/v) Triton X-100 (3). Lower panel, left part: ‘free’ activities of urate oxidase in peroxisomes from livers of control (dark gray column) and *Pxmp2* ^−/−^ (light gray column) mice. *P = 0.0008, n = 12; Lower panel, right part: total activity of urate oxidase in ‘postnuclear’ liver homogenates. (E) Determination of uric acid in blood (left panel, male mice) and urine (right panel, female mice) of wild-type (•) and *Pxmp2^−/−^* (○) mice. Similar results were obtained with mice of both gender types. The excretion of uric acid and allantoin (see below) into urine is presented as molar ratios to creatinine (see ‘Methods’ for details). ^*^P = 0.010; ^**^P = 0.008. (F) Allantoin content in blood (left panel) and urine (right panel) of wild-type (•) and *Pxmp2^−/−^* (○) mice. *P = 0.067.

To elucidate biological roles of Pxmp2, we inactivated the *Pxmp2* gene by homologous recombination in mouse embryonic stem cells (Figure 1A and Figure S1) and generated heterozygous *Pxmp2* ^+/−^ mice. Crossing of *Pxmp2* ^+/−^mice gave *Pxmp2* ^−/−^ progeny at the predicted Mendelian frequency. They showed normal postnatal development, displayed no detectable morphological tissue abnormalities in gross examination and were fertile. However, the female *Pxmp2^−/−^* mice encountered difficulties in puerperant nursing of pups (see ‘Discussion’ for details). The levels of tested peroxisomal proteins (catalase, 3-oxoacyl-CoA thiolase, sterol carrier protein 2/3-oxoacyl-CoA thiolase, and sterol carrier protein 2) were similar in immunoblots of liver samples from either wild-type, Pxmp2^+/−^ or *Pxmp2* ^−/−^ mice (data not shown). The Pxmp2 deficiency did not affect activities, measured in liver homogenates, of the enzymes confined to peroxisomes: catalase, carnitine acetyltransferase, carnitine octanoyltransferase, and L-α-hydroxyacid oxidase (data not shown). Electron microscopy revealed that size and shape of liver peroxisomes from *Pxmp2* ^−/−^ mice was normal (Figure 1B).

We used Nycodenz gradients to separate Pxmp2-deficient liver peroxisomes from other cellular components and detected an increase in the leakage of soluble matrix proteins from the particles from *Pxmp2* ^−/−^ mice relative to those from wild-type animals (Figure 1C and Figure S2A, S2B, S2C, S2D). The isolated Pxmp2-deficient peroxisomes contained matrix with a lower electron density as compared to control preparations (Figure 1B and Figure S2E). The results obtained indicate an elevated fragility of Pxmp2-deficient peroxisomes which can be prevented *in vitro* using polyethylene glycol (PEG) 1500 as an osmoprotectant (Text S2 and Figure S2F, S2G, S2H). These observations suggest abnormal osmotic behavior by the particles due to apparent limitations in the membrane permeability to solutes (Figure S2I, S2J).

Unlike catalase and cofactor-dependent enzymes, cofactor-independent peroxisomal oxidases, such as urate oxidase, show no latency in peroxisomes from wild-type rodents [5], [20]. However, activities of urate oxidase and L-α-hydroxyacid oxidase in the liver peroxisomes from *Pxmp2* ^−/−^ mice displayed latencies (Figure 1D, Text S3, and Figure S3A). Only a part of the ‘total’ activities of the oxidases was latent, whereas activities of catalase and cofactor-dependent enzymes measured in the same peroxisomal preparations showed high latency. The results demonstrate that the Pxmp2 deficiency leads to partial restriction in the peroxisomal membrane permeability to metabolites *in vitro*.

The restricted diffusion of substrates into peroxisomes might decrease their rate of metabolism by the corresponding peroxisomal enzymes such as, for example, oxidation of uric acid to allantoin by urate oxidase, which is present in mouse only in the liver (Text S3 and Figure S3B, S3C, S3D). To test this possibility we measured the steady-state concentrations of uric acid and allantoin in mouse body fluids. The results showed elevated levels of uric acid in blood and increased clearance with urine in *Pxmp*2^−/−^ mice compared to wild-type animals (Figure 1E). Concomitantly, a decrease in allantoin excretion suggests that the elevated levels of uric acid observed are due to a decrease in the degradation of this compound rather than due to enhancement in purine catabolism in *Pxmp*2^−/−^ mice (Figure 1F). Because the ‘total’ urate oxidase activity (Figure 1D) and the content of urate oxidase protein (Figure S3E) were the same in liver homogenates of *Pxmp*2^−/−^ and wild-type animals, the results agree with the notion that the decreased uric acid catabolism is due to restriction in peroxisomal membrane permeability. Results supporting this suggestion were obtained using mice challenged by administration of glycolic acid. Decreased metabolism of this compound in peroxisomes, owing to a restriction in peroxisomal membrane permeability, may lead to elevated formation of oxalic acid. The experimental data confirm this supposition (Figure S3F).

### Channel-forming activities in peroxisomes of Pxmp2-deficient mice

**Figure 2:**
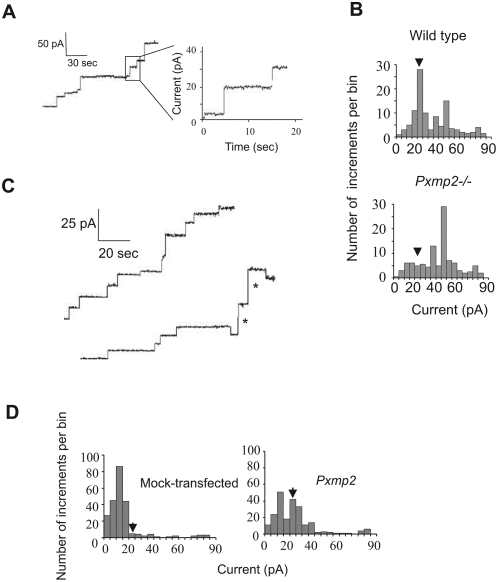
Detection of pore-forming activity in membrane preparations. (A) Traces of the multiple-channel recording of an artificial membrane in the presence of detergent-solubilized liver peroxisomes. The right trace shows a timescale-expanded current recording from the left trace. The current measurements (Figure 2A–2D and Figure 3B and 3C) were made with 1.0 M KCl on both sides of a bilayer and at applied voltage +20 mV. (B) Histogram of multiple-channel recordings registered in a peroxisomal fraction isolated from livers of wild-type (upper panel) or *Pxmp2* ^−/−^ (lower panel) mice. The total number of insertion events: upper panel - 213; lower panel - 210. Here, and on Figure 2D each experiment was repeated at least three times and typical pictures are presented; the 1.3 nS conductance level is marked by an arrowhead on each panel. (C) Multiple-channel recordings of PPF preparation isolated from mock-transfected (upper trace) insect cells or those expressing recombinant Pxmp2 (lower trace). Insertion events with a conductance of 1.3 nS in 1.0 M KCl are marked by asterisks. (D) Histogram of insertion events observed in the presence of PPF preparations isolated from mock-transfected (left panel) and Pxmp2-containing (right panel) insect cells. The total number of insertion events: left panel - 230; right panel - 225.

Our results can readily be explained by the presence of general diffusion channels/pores in peroxisomal membrane which are formed by the Pxmp2 protein. Therefore, using a reconstitution assay in lipid bilayers, we analyzed channel-forming activities in peroxisomes isolated from livers of wild-type and *Pxmp2* ^−/−^ mice. In agreement with previous observations [6], the peroxisomal membrane preparations from wild-type mice showed the most frequent insertion events with an average conductance of 1.3 nS and 2.5 nS in 1.0 M KCl (Figure 2A and 2B). When peroxisomal membranes of *Pxmp2* ^−/−^ mice were analyzed, the fluctuations with a conductance increment of 1.3 nS in 1.0 M KCl were lost.

### Expression of Pxmp2 in insect cells

Mouse Pxmp2 was expressed in the insect cell line Sf9 using a baculovirus expression system. Immunodetection of Pxmp2 indicated that the recombinant protein is concentrated in the postmitochondrial particle fraction (PPF) containing microsomes and (micro)peroxisomes (Figure S4A, S4B). Multiple-channel recording of the PPF isolated from mock-transfected cells revealed several types of endogenous channel-forming activity. Pxmp2-expressing cells showed an abundant channel-forming activity with a conductance of 1.3 nS in 1.0 M KCl (Figure 2C and 2D). This activity was suppressed by antibodies raised against Pxmp2 (Figure S4C). Proteins from the PPF containing recombinant Pxmp2 were solubilized and fused to a planar bilayer for a single-channel analysis (see ‘Methods’ for details). We identified a channel with the same conductance (1.32±0.20 nS slope conductance, 1.0 M KCl, n = 4), cation selectivity (E_rev_ = 6.8 mV, 1.0/0.5 KCl, P_K+_/P_Cl−_≈2.35), and voltage dependence as the channel formed by purified Pxmp2 (see next section). Collectively, these results indicate that the recombinant Pxmp2, like its native counterpart, shows channel-forming activity.

### Channel-forming activity of purified Pxmp2

**Figure 3:**
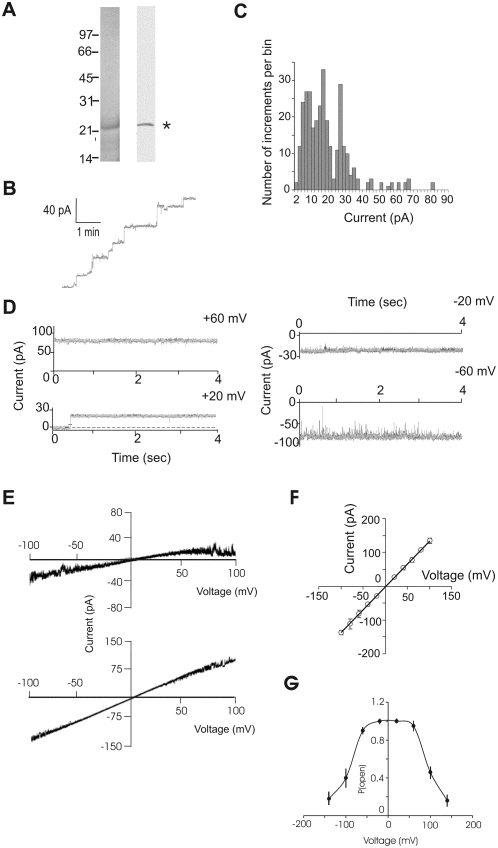
Channel-forming activity of the purified Pxmp2. (A) SDS-PAGE and immunoblot analysis of the purified native Pxmp2. Left panel: fractions obtained after the final purification step (second size-exclusion chromatography step, see Text S1) and which were enriched with Pxmp2, were concentrated and proteins (∼20 ng) were subjected to SDS-PAGE followed by silver staining. Right panel: immunoblot analysis of the purified Pxmp2. The Pxmp2 band is marked by an asterisk. (B) Multiple-channel recording of a bilayer after addition of purified Pxmp2 (∼10 ng/ml, final concentration). (C) Histogram of insertion events of purified Pxmp2. The total number of increments is 312. (D) Current traces from a bilayer containing single Pxmp2 pore-forming protein (the insertion event shown on the left panel, the lower trace) at different membrane potentials (1.0 M KCl on both sides of the membrane). Applied membrane potentials are indicated. The data were filtered at 1.0 kHz and recorded at 2.0 kHz. (E) Current traces of low- (upper panel) and high (lower panel) conductance channels in response to voltage ramp protocol (from −100 to 100 mV, 10 sec). 1 M KCl on both sides of the membrane. (F) Current-voltage relationship of the fully open (25–30 pA at +20 mV, 1.0 M KCl) single channel (averages of six independent bilayers±S.D.). (G) Voltage dependence of the probability (P_open_) of the high-conductance Pxmp2 channel being in a fully open state. After application of different voltage steps at t = 0, mean currents were calculated for a time period of 40 sec (from t = 20 sec to t = 60 sec). Data were normalized to the maximal currents of the fully open state at the corresponding holding potentials. Data points are an average of n = 4 independent bilayers.

To obtain conclusive evidence that Pxmp2 forms a channel the native protein was isolated from mouse liver peroxisomes (Text S1, Figure 3A, and Figure S5). Multiple channel recordings using this protein showed pore-forming activities mainly at three conductance levels: 0.45 nS, 0.9 nS, and 1.3 nS in 1.0 M KCl (Figure 3B and 3C). The conductance of both low- (0.45 nS) and high- (1.3 nS) conductance channels depended nearly linearly on the KCl concentration (Figure S6A).

**Figure 4:**
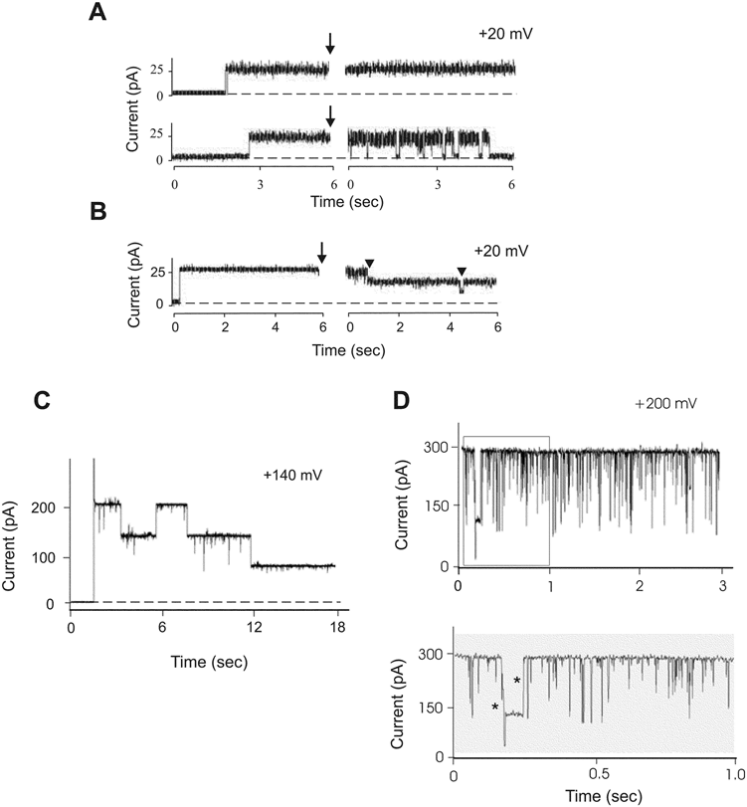
Sub-conductance states of the Pxmp2 channel. (A) Effect of anti-Pxmp2 antibodies on the pore-forming activity of Pxmp2. The results presented on panels A–D were collected on Pxmp2 channels at fully open state; the data were filtered at 0.4 kHz (A and B) or at 1.0 kHz (C and D) and recorded at 2.0 kHz. Single Pxmp2 channel was incorporated into lipid bilayer and analyzed before and after addition (marked by arrow) of the IgG fraction (3 µg) isolated from pre-immune (upper trace) or immune (lower trace) serum to both compartments of the chamber. The time gap is 120 sec. Here, and on panel B, experiments were repeated 6 times, typical pictures are presented. (B) Effect of alkalization of the bath solution on the pore-forming activity of Pxmp2. Current traces of a bilayer containing one Pxmp2 pore-forming protein before and after addition (marked by arrow) of 0.2 M Na_2_CO_3_, pH 11.2 (150 µl) to both compartments of the chamber are shown. The time gap is 60 sec. Two steps of the channel closure are marked by arrowheads. (C) High holding potential leads to closure of the Pxmp2 channel. The artificial membrane contained a single high-conductance channel (the insertion event not shown). Note stepwise closure of the channel at high holding potential, the amplitude of each step constitutes one third of that one of the fully open channel. (D) Flickering of a single Pxmp2 channel incorporated into lipid bilayer. The lower trace shows a timescale-expanded recording from the part (in the frame) of the upper trace. The direct transitions between three main subconductance states are marked by asterisks.

In a separate set of experiments we conducted a single-channel analysis of the purified Pxmp2 protein. Fusion events yielding insertion of one channel were monitored and pore-forming activities recorded at different potentials (Figure 3D). During short exposure (less than 30 sec) to membrane potentials in a range of V_m_ = ±100 mV, the channels were mainly open although at potentials above V_m_ = ±60 mV brief, flickering closures of the channels were frequently observed. With 1.0 M KCl bath solution on both sides of the membrane, the low- and high-conductance channels showed a near linear current-voltage relationship in response to a rapidly increasing voltage ramp (Figure 3E). The calculated slope conductance of the fully open channel was 1.34±0.16 nS (Figure 3F). Figure 3G shows the voltage dependence of the high-conductance channel open probability during prolonged application of a constant voltage. In a range of membrane potentials V_m_ = ±60 mM the channel was near completely open, whereas it stepwise closed at more positive or negative membrane potentials with transition to intermediate- and low-conductance states (see below). Thus, the Pxmp2 channel closes slowly during prolonged exposure to elevated membrane potentials. In asymmetric bath solutions (1.0 M KCl/0.5 M KCl) the reversal potential was E_rev_ = +6.5 mV for the high-conductance single channel (Figure S6B, S6C). Accordingly, the channel is moderately cation-selective (P_K+_/P_Cl−_ = 2.3). A very similar value (P_K+_/P_Cl−_ = 2.4) was detected when the measurements were made on a low-conductance channel (0.45 nS in 1.0 M KCl, data not shown). Addition of antibodies against Pxmp2 into the bath solutions during current recordings of a single fully open channel led to its closure (Figure 4A).

A spontaneous, voltage-independent transition of the 1.3 nS channel to other states was occasionally observed during multiple-channel recordings (data not shown). The sub-conductance states of the channel were always close to 0.45 nS and 0.9 nS in 1.0 M KCl. To verify presence of the sub-states by means of a single-channel analysis we treated the high conductance channel inserted in the bilayer at alkaline pH and observed a stepwise closure of the channel with each step showing a conductance of 0.45 nS in 1.0 M KCl (Figure 4B). Likewise, a stepwise closure of the high-conductance channel at holding potentials V_m_ = ±100 mV or higher with two sub-conductance levels was observed (Figure 4C). Similarly, short exposure to high potentials (V_m_≥150 mV) led to a frequent appearance of the high-conductance channels (1.3 nS in 1.0 M KCl) that showed flickering closure sometimes with evident sub-conductance levels of 0.9 nS and 0.45 nS in 1.0 M KCl, respectively (Figure 4D). The transition of the high-conductance channel into sub-states can be interpreted in terms of a cluster of three small channels, each of them with a conductance around 0.45 nS in 1.0 M KCl (see ‘Discussion’ for more details).

### Permeability properties of the Pxmp2 channel

We performed additional reconstitution assays in lipid bilayers in order to obtain information on the size of the channels formed by Pxmp2 (Text S1 and Figure S7A, S7B, S7C). From these experiments we concluded that the radius of the narrowest space of the channel (channel friction) is about 0.7 nm. Such a channel allows nearly free diffusion of ions and non-electrolytes with molecular masses of up to 200–300 Da across the membrane. The movement of larger molecules, from 300 Da to 600 Da in size, is limited, while more bulky solutes are unable to permeate the membrane through the channel.

**Figure 5:**
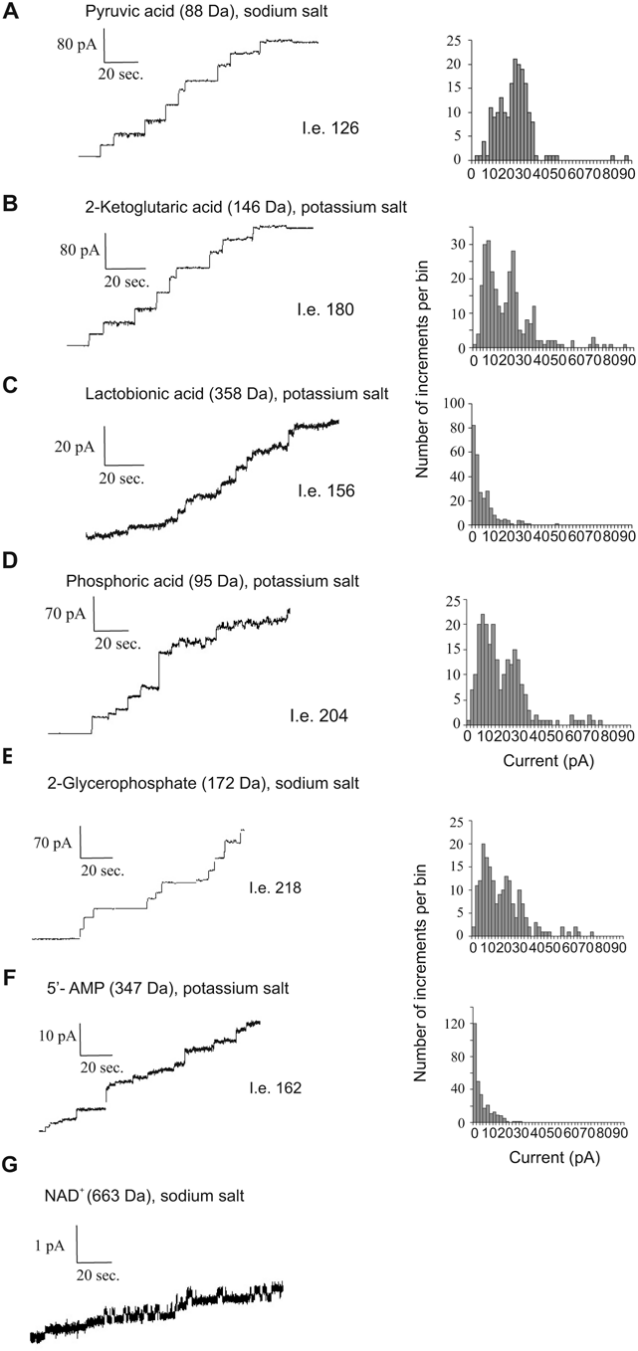
Multiple-channel recording of purified Pxmp2 using different organic anions as electrolytes. The measurements were made using as a bath 1.0 M solutions of potassium or sodium salts of the anions at pH 7.2. The pH of solutions was adjusted by corresponding sodium or potassium hydroxides. The solutions were buffered with 10 mM MOPS, pH 7.2. Phosphate was used as 1 M potassium phosphate buffer, pH 7.2. Molecular masses of the corresponding anions are shown in brackets. The total number of insertion events (I.e.) is also shown. Two different batches of purified Pxmp2 were used with similar results obtained; typical pictures are presented. The purified Pxmp2 channel was also active with glycolic, lactic, acetic and allantoic acids (data not shown). We did not analyze in depth the dependence between size of anions and their conductance level since the hydrated radii of most of these anions are not known. However, as can be seen from the data, the conductivity of the Pxmp2 channel is clearly dependent on the size of the anions if their molecular mass exceeds 300 Da (compare, e.g., panels A and B with panels C and F), indicating partial restriction in the diffusion of these anions through the channel.

To further analyze the function of the Pxmp2 channel we made direct measurements of the pore-forming activity of purified protein using various organic anions as electrolytes (Figure 5A–5G). A variety of small mono- and divalent anions known to be peroxisomal metabolites, such as glycolate, pyruvate, 2-ketoglutarate, and others, can be transferred through the Pxmp2 channel. As expected, if the size of the anion is over 300 Da, e.g, lactobionic acid (358 Da) or AMP (347 Da), the single-channel conductance of Pxmp2 is significantly decreased, indicating that the movement of these compounds inside the channel is partially restricted. The only traces of channel-forming activity with a conductance well below 20 pS were detected in the presence of NAD (663 Da). No channel-forming activities were observed with ATP (507 Da), probably due to the high net negative charge of this molecule, which prevents diffusion of ATP through the cation-selective Pxmp2 channel.

## Discussion

In our study we tried to resolve two important interrelated problems in the physiology of mammalian peroxisomes: (i) the molecular foundation for the permeability of the peroxisomal membrane to solutes, and (ii) the functional role of Pxmp2, which belongs to a membrane protein family with a previously unknown function.

### Pxmp2 forms a channel in peroxisomal membrane

According to our recent observation [6], the putative peroxisomal channels show single channel conductance of 1.3 nS and 2.5 nS in 1.0 M KCl, respectively, when crude peroxisomal membrane preparations from mouse liver were assayed in reconstitution experiments using lipid bilayers. The present work demonstrates that Pxmp2 is responsible for one of the described channel-forming activities, namely for the activity with a conductance of 1.3 nS in 1.0 M KCl. The evidence that Pxmp2 forms a channel is at least five-fold: (i) knocking out of *Pxmp2* leads to partial restriction of peroxisomal membrane permeability to small solutes *in vitro* and *in vivo*; (ii) the pore-forming activity with a characteristic conductance of 1.3 nS in 1.0 M KCl was not observed in the peroxisomal membrane preparations isolated from the livers of Pxmp2- deficient mice; (iii) expression of recombinant Pxmp2 in insect cells resulted in the appearance of a pore-forming activity with a conductance of 1.3 nS in 1.0 M KCl; (iv) this activity was inhibited following treatment of solubilized membrane proteins with antibodies generated against Pxmp2; and (v) isolated Pxmp2 showed pore-forming activity with three conductance levels, the highest one being 1.3–1.4 nS in 1.0 M KCl.

### Does peroxisomal membrane open to small solutes?

The controversy of the area (see Introduction) is exemplified by two recent studies of the apparent role of mammalian peroxisomes in Ca homeostasis published ‘back-to-back’ in the same journal [11], [12]. The data concerning pH gradients across peroxisomal membrane are even more confusing. For instance, in one report the authors made a conclusion that the matrix of mammalian peroxisomes is basic [9]. However, another study revealed that mammalian peroxisomes have no cross-membrane pH gradient at all [10]. The results obtained on yeast peroxisomes are also contradicting: some reports claim basic pH in the particles [21] while other publications indicate acid pH in the same organelles [22].

One explanation for difference in the results described above is the possible existence of Donnan equilibrium between peroxisomal matrix and cytoplasm surrounding the particles. In this case the formation of pH or Ca gradients does not require membrane impermeable to small ions. Instead, the gradients are formed across the membrane permeable to solutes by difference in overall charges of molecules unable penetrate this membrane (e.g., proteins) which are localized inside and outside the particles [23]. For example, if the overall charge of proteins inside peroxisomes is more positive than outside the particles, these proteins should attract small negatively charged solutes, including hydroxyl ions, to preserve electroneutrality. As a result, the pH gradient is formed, where pH inside peroxisomes is more basic than outside the particles. The mechanism of Donnan equilibrium depends on free permeation of small charged solutes, including protons and hydroxyl ions, across the membrane [23]. The Donnan-type equilibrium may be responsible for creation of a pH gradient across outer mitochondrial membrane [24] and involved in the maintenance of an acid pH in lysosomes [25].

An apparent role of Donnan equilibrium in creation of ion gradients across peroxisomal membrane open to small solutes is consistent with numerous observations collected within last 40 years by different groups showing that mammalian peroxisomal membrane does not form a barrier to these solutes *in vitro* (see, e.g., ref. [5], [19], [20], [26]). Our data obtained on peroxisomes isolated from livers of Pxmp2-deficient mice are in line with this conclusion. Moreover, these data revealed direct involvement of the Pxmp2 channel in the transfer of solutes across the membrane. Likewise, the results of our study of peroxisomal metabolites (urate, oxalate) in body fluids of Pxmp2-deficient mice corroborate *in vitro* findings.

### Apparent functions of the Pxmp2 channel

Our results predict that the Pxmp2 protein forms a relatively wide, water-filled channel in which the mobility of small solutes is determined by their diffusion coefficients. This implies that the Pxmp2 channel is non-selective with respect to the chemical nature of solutes, whereas it is highly selective relative to the size of solutes. In addition, because of its very long open states the Pxmp2 behaves like a pore similar to porins of outer mitochondrial membrane or outer membrane of gram-negative bacteria. These features of the channel apparently determine the unusual permeability properties of peroxisomal membrane, resulting in a novel type of biomembrane exploiting both: pore-forming proteins as well as solute transporters (e.g., ATP transporter [4]) to transfer metabolites in and out of peroxisomes. The present results combined with our previous observations [5] point to the ability of peroxisomal membrane to discriminate between small metabolites with sizes typically below 200 Da and ‘bulky’ solutes including ATP and cofactors (NAD/H, NADP/H, CoA and its acylated derivatives) (Figure S7D). It appears that the large molecular size of cofactors and some other solutes is an important factor determining their subcellular localization.

**Figure 6:**
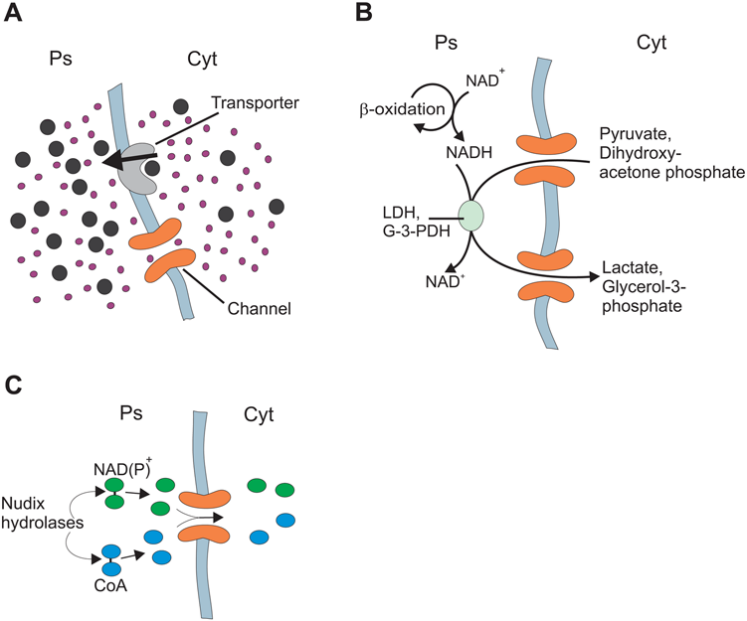
Transfer of solutes across peroxisomal membrane. (A) Cooperation of non-selective channels and selective transporters in transfer of small (○) and ‘bulky’ (•) solutes across the peroxisomal membrane. Ps, peroxisomal lumen; Cyt, cytoplasm. The arrow indicates the vectorial transportation of the ‘bulky’ compound leading to formation of a separate pool for this solute in the peroxisomal lumen. (B) An apparent role of peroxisomal channels in the transfer of shuttle molecules participating in regeneration of NADH produced by the β-oxidation of fatty acids in peroxisomes. LDH: lactate dehydrogenase, G3PDH: NADH-dependent glycerol-3-phosphate dehydrogenase; both enzymes are partially localized to peroxisomes. (C) An apparent role for Nudix hydrolases in removal of cofactors out of peroxisomes (see ‘Discussion’ for details).

Given that the Pxmp2 channel does not restrict permeation of small solutes, it is possible that peroxisomes share a common pool of these solutes with the surrounding cytoplasm (Figure 6A). In contrast, the ‘bulky’ solutes are unable penetrate the membrane through channels and require specific transporters, which may generate gradients of these solutes between peroxisomal lumen and cytoplasm.

How the peroxisomal metabolic machinery may exploit the Pxmp2 channel to carry out some specific functions is easy to predict. It is generally accepted that the metabolic conversion of peroxisomal cofactors proceeds via shuttle mechanisms resembling such systems in the inner mitochondrial membrane [27]. However, in contrast to mitochondria, the shuttle molecules in peroxisomes do not need specific transmembrane transporters owing to the presence of pore-forming proteins in the membrane (Figure 6B).

Several members of the family of Nudix hydrolases are located in peroxisomes and active towards cofactors (CoA, NAD/P), cleaving them into two parts of near equal size [28], [29]. The reaction leads to formation of molecules which are able to cross the membrane using peroxisomal channels and it provides a route for the removal of cofactors from peroxisomes (Figure 6C). Thus, cleavage of NAD^+^ (663 Da) by the corresponding peroxisomal Nudix hydrolase NUDT12 produces NMNH (334 Da) and AMP (347 Da). Permeation of NAD^+^ through the Pxmp2 channel is negligible (see Figure 5G). However, the channel is permeable to AMP and apparently also to NMNH based on the similar size of these molecules (NMNH has no net charge, preventing use of this compound for multiple-channel recording).

### Pxmp2-deficient mice

The *Pxmp2* knock-out mouse model allowed us to collect additional evidence for the presence of at least two types of channels in mammalian peroxisomes: (i) the Pxmp2 deficiency led to only partial restriction in peroxisomal membrane permeability to solutes *in vitro*; (ii) the increase in the content of uric acid in the serum and urine of *Pxmp2* ^−/−^ mice relative to wild-type control, being 1.4-fold and 3.1-fold, respectively (see Figure 1E), was much lower than that seen for urate oxidase-deficient mice, where the corresponding levels of uric acid were 10-fold and 9-fold higher than in the wild-type animals [30]. The last observation indicates that in spite of the absence of Pxmp2, a significant amount of uric acid is still degraded in peroxisomes (as also indicated by excretion of allantoic acid into urine) and suggests the existence of a second peroxisomal transmembrane route for this metabolite; (iii) solubilized membrane preparations from Pxmp2-deficient peroxisomes still displayed the pore-forming activity with a wide range of peroxisomal metabolites as electrolytes (data not shown).

The mild phenotype of Pxmp2-deficient mice may reflect redundancy of the peroxisomal channels in their ability to transfer solutes across the membrane. The redundancy of the function of mammalian peroxisomal proteins, at least at normal physiological conditions, is a well known phenomenon. The examples of poor phenotype of the mouse models deficient in certain peroxisomal protein are numerous and include multifunctional enzyme type 1 [31], racemase [32], liver isoform of fatty acid binding protein [33], and peroxisomal membrane ABC transporters (reviewed in [34]). The functional redundancy might be limited or even abolished by harsh environmental conditions. To examine this possibility we challenged *Pxmp2^−/−^* mice with diets containing clofibrate (a known proliferator of mouse liver peroxisomes) or phytol (methyl-branched fatty alcohol metabolized via α-oxidation only in peroxisomes). These treatments did not trigger development of phenotypes in Pxmp2-deficient mice different from those of wild-type animals (data not shown). However, our in depth analysis of the effect of Pxmp2 deletion on female mice reveals some unexpected results. In addition to disturbances in uric and oxalic acids metabolism (see above), these mice were unable to nurse their pups due to low production of milk. *Pxmp2^−/−^* female mice showed retarded growth of mammary glands and limited abnormalities in reproductive organs during pregnancy (Rokka A. and Vapola M, unpublished results). It's not yet clear how Pxmp2 deficiency affects mechanisms responsible for normal development of mammary glands. This specific problem is under of our current investigation.

### Cluster organization of the Pxmp2 channel

To describe the nature of the sub-conductance levels of the isolated Pxmp2 channel, one would have to discriminate between two possibilities: (i) whether the large channel represents a cluster of small channels as in the case of some antibiotics forming membrane pores [35] or bacterial porins [36] or (ii) whether the sub-conductance states are the result of channel gating, as was shown for the mitochondrial voltage-dependent anion channel (VDAC) [37]. The transition of the high conductance level (1.3 nS in 1.0 M KCl) to lower conductance levels (0.9 nS and 0.45 nS in 1.0 M KCl, respectively) can be observed (see Figure 4B–4D). If this last observation were to reflect the closure of the channel, it would then be reasonable to expect: (i) the pore radii of the large and small channels should be different, and (ii) cation-anion selectivity of the channel would be affected by the closure event, as was shown for mitochondrial VDAC [37]. If large conductance channels represent clusters of smaller ones, then both of them should demonstrate very similar size and ion selectivity. Our data showing high similarity in ion selectivity and predicted size between Pxmp2 channels with different conductance rates favor the cluster organization model for the channel.

In view of the homotrimeric composition of Pxmp2 (see Text S1 and Figure S5), it would be reasonable to predict that each subunit of the protein cluster forms a channel on its own. This architecture, a cluster of three identical monomers, each forming a discrete transmembrane pore, is not unique, since most bacterial porins [36] and the preprotein translocation channel (TOM complex channel) of the outer membrane of mitochondria [38] show similar multimeric assemblies. Interestingly, the freshly isolated TOM complex shows three conductance levels. However, after sonication or multiple freeze-thaw cycles, channels having only two main conductance levels were observed, prompting the authors to suggest that physical treatments may lead to inactivation of one of the pores in the complex [38]. This observation resembles our finding indicating three conductance levels for the isolated Pxmp2 channel instead of one conductance level of around 1.3 nS in 1.0 M KCl as would have been expected from the data obtained using peroxisomal membranes from Pxmp2 deficient mice and membranes from transfected insect cells.

The results presented here demonstrate a function for Pxmp2 and predicts a mechanism by which water-soluble metabolites penetrate the peroxisomal membrane. The overall transport function of this membrane is derived from exploitation of pore-forming proteins and transporters specific for certain metabolites. This arrangement of two different transport systems in one membrane may not be limited only to the mammalian peroxisomes, but it could be a property of the other members of the microbody organelle family. For instance, glycosomes of trypanosomatids, unicellular parasites that cause sleeping sickness in humans, contain almost the whole set of glycolytic enzymes and conduct several functions attributable to mammalian and yeast peroxisomes [39]. The low diversity of glycosomal membrane proteins [40] suggests that these organelles may rely on a transmembrane transfer mechanism similar to that of mammalian peroxisomes, avoiding the requirement for specific transporters while preserving the tight regulation of glycolysis that, as has been shown [41], is vitally important for survival of the parasite in erythrocytes of host mammals.

## Materials and Methods

### Targeted disruption of Pxmp2 in mice

The mouse *Pxmp2* gene is located head to head with the *PoleI* gene encoding the catalytic subunit of DNA polymerase **∈**. The translation initiation codons of these two genes are separated by only 393 bp. *Pxmp2* and *PoleI* have been shown to have independently regulated expression [42]. To produce Pxmp2 deficiency, a targeting vector was constructed such that after homologous recombination of the disruption cassette at exon 2 of *Pxmp2*, a 2.7 kb segment flanking the region 5′ of the translation initiation code in *PoleI* remained intact (Figure S1A). This strategy was chosen so as not to alter any regulatory elements or impact the expression of *PoleI*.

To disrupt *Pxmp2* a LacZ-PGKneo cassette (see below) was inserted into exon 2 and the disruption was verified by partial sequencing of the exon/intron junctions. The restriction map of the *Pxmp2/PoleI* structure was a gift of Prof. J. Syväoja (University of Oulu, Finland). The BAC ES-clone containing *Pxmp2* was obtained from GenomeSystems. A 1.2-kb fragment spanning the region from the *EcoR*I site downstream of exon 1 to the *Xba*I site in exon 2 was generated from the *Pxmp2* genomic BAC ES-clone for use as the 5′-homology arm of the targeting construct, and subcloned into a pBluescript II SK vector (Stratagene). A LacZ-PGKneo cassette containing the *lacZ* reporter gene without an ATG start codon and the neomycine-resistance gene for positive selection (PGKneo) in head to head transcriptional orientation, was ligated downstream of the 5′-homology arm. As the 3′ homology arm, a 4.7 kb *Xba*I-*BamH*I fragment containing the second half of exon 2, exon 3 and intron 3 was inserted downstream of the LacZ-PGKneo cassette.

The targeting vector was linearized with *Not*I and electroporated into 129/SvJ RW4 embryonic stem (ES) cells. After 24 h cell growth, 200 µg/ml G418 (GIBCO/BRL) was added to the medium and the neomycine selection was carried out for 4–5 days. The surviving ES-cell colonies were screened for the correct insertion of the LacZ-PGKneo cassette by PCR using 3 primers (Figure S1A): (1) the forward primer for wild-type and mutated alleles (GGTCAGAAGCACAGAGAAGAGAAGC) corresponding to the sequence from intron 1, upstream of the 5′-flanking region; (2) the reverse primer for the wild-type allele (CGCCCAGCTTCTCTGATGCTTCTTA) from intron 2, and (3) the reverse primer for the mutated allele (GCGGGCCTCTTCGCTATTACG) from the *lacZ*-reporter gene. The sizes of the PCR products generated were 1.7 kb and 1.5 kb, corresponding to the wild-type and targeted alleles, respectively. Positive ES-cell clones were verified by Southern blotting (see below). Recombinant (*Pxmp2* ^+/−^) ES-cell clones were used for aggregation with C57BL6/J morulas. The resulting chimeras were mated with C57BL/6J mice. The *Pxmp2* ^+/−^ germline offsprings (F1 generation) identified by PCR analysis of tail-tip genomic DNA, were backcrossed with C57BL/6J mice for 7 generations.

The disruption of *Pxmp2* was verified by Southern blot analysis (Figure S1B). Northern blotting (Figure S1C) and quantitative real-time PCR (data not shown) confirmed the absence of *Pxmp2* transcripts. The inactivation of *Pxmp2* was further demonstrated by immunodetection of the corresponding protein in liver homogenates from wild-type, *Pxmp2* ^+/−^ and *Pxmp2* ^−/−^ mice (Figure 1A). The proximity of *PoleI* to *Pxmp2* prompted us to investigate the expression of this gene in spleen, a tissue with a high rate of cell proliferation characterized by a substantial level of *PoleI* expression [42]. The results of quantitative real time PCR showed no difference in the expression levels between wild-type and Pxmp2-deficient mice, indicating that the expression of *PoleI* was not affected by the disruption of *Pxmp2*.

### Expression of recombinant Pxmp2 in insect cells

Mouse kidney total cDNA was used to amplify *Pxmp2* cDNA by PCR with the forward and reverse primers (CCG**GAATTC**ACCATGGCAACCTGCGGG **and CCG** **GAATTC**TCACTTCCCCAGAGACC, respectively) containing the *EcoR*I restriction sites (shown in bold). The blunt-ended PCR products were cloned into the *Sma*I site of pUC18 vector (Amersham). The BAC-TO-BAC™ Baculovirus Expression System (Invitrogen) was used for generating of recombinant baculovirus and transfection of Sf9 insect cells. Mock-transfection was performed with recombinant baculovirus containing the gene coding for human lysyl hydroxylase (gift of Prof. R. Myllylä, University of Oulu, Finland).

The infected cells were homogenized in 20 mM MOPS, pH 7.2 containing 0.25 M sucrose and 1 mM EDTA. The homogenate was centrifuged at 800 g_max_ for 10 min to remove nuclei and cell debris. The resulting postnuclear supernatant was centrifuged at 100,000 g_max_ for 45 min to obtain the total membrane fraction and the cytosol. The postmitochondrial particles fraction (PPF) was isolated by centrifugation of the post-nuclear homogenate at 6000 g_max_ for 20 min and the resulting supernatant was centrifuged at 100,000 g_max_ for 45 min. The compositions of the isolated fractions were determined using marker enzymes for different subcellular organelles.

### Southern and northern analysis

For Southern analysis the genomic DNA was extracted from mouse liver using a Blood and Cell Culture Midi Kit (Qiagen). 12 µg of DNA was digested with *Sac*I restriction enzyme and hybridization of the blot was carried out at 65°C using a ^32^P-labeled (Random Primed Labeling Kit, Amersham) 670 bp external probe which corresponds to the sequence of intron 1 upstream of the 5′ flanking region (Figure S1A). For Northern analysis, total RNA was isolated from mouse liver using a Quick Prep Total RNA Extraction kit (Amersham). 45–50 µg of RNA was separated using agarose gel electrophoresis and blotted for hybridization with full-length mouse Pxmp2 cDNA.

### Subcellular fractionation and isolation of mouse liver peroxisomes

The use of experimental animals was approved by the committee on animal experimentation at the University of Oulu. Male or female C57BL/6J mice were used. In some experiments mice were maintained 12 weeks on a standard diet containing 0.5% (w/w) phytol (Aldrich) or two weeks on a diet containing 0.5% (v/w) clofibrate (Aldrich). Peroxisomes were isolated using density gradient centrifugation technique as described in details in Text S1.

### Measurement of enzyme activities and latency determination

Enzyme activities and latency were detected by spectrophotometric assay as described in Text S1.

### Isolation of native Pxmp2 and characterization of oligomeric structure

The Pxmp2 protein was isolated from mouse liver peroxisomes using conventional chromatography technique. The oligomeric structure of the Pxmp2 protein was analyzed by means of size-exclusion chromatography and cross-linking experiments. See Text S1 for details.

### Estimation of the pore size of the Pxmp2 channel

The pore diameter of isolated Pxmp2 channel was estimated by means of electrophysiological technique using monovalent cations as electrolytes or concentrated solutions of non-electrolytes (mainly polyethylene glycols with different hydrated radii). See Text S1 section for details.

### Electrophysiological measurements

Multiple-channel recordings and single-channel analysis of Pxmp2 protein were performed using Planar Lipid Bilayer Workstation equipped with a BC-535 amplifier and a 8 pole low-pass Bessel filter (Warner Instruments). Acquisition and analysis were performed using the pCLAMP software (Axon Instruments).

Multiple-channel recordings were performed as described previously [38] with some modifications. The artificial membrane was formed by means of painting techniques using 1% (w/v) diphytanoyl phosphatidylcholine (Avanti Polar Lipids), dissolved in n-decane/butanol (9∶1, v/v). Membrane formation occurred across a circular hole (0.2 mm^2^) in the thin wall separating two compartments (5 ml each) in a Teflon chamber. The resulting bilayers had a typical capacitance of 300–700 pF. The aqueous salt solutions (analytical grade) were unbuffered (unless otherwise stated) and had a pH of around 6. Membrane proteins were solubilized in 0.5% (w/v) Genapol X-080 (Fluka) by rotating for 1 h at +4°C. An insoluble material was sedimented by centrifugation at 100,000 g_max_ for 45 min and the resulting supernatant was immediately used for detection of the pore-forming activity. Purified Pxmp2 protein was dissolved in 0.5% (w/v) n-dodecyl-β-D-maltoside (Sigma). Peroxisomal channels were inserted into the lipid bilayer at high frequency in 1.0 M KCl bathing solution [6] and this salt concentration was used in all experiments unless stated otherwise. Solubilized membrane proteins or purified Pxmp2 protein preparations (4 µl) were added to both compartments of the chamber for incorporation into the bilayer, which occurred spontaneously within 5–10 min. The temperature was maintained at 20°C. Control experiments did not reveal any spontaneous channel-like activity in the presence of the detergent only. Membrane currents were measured at a membrane potential of +20 mV (unless otherwise stated) with a pair of Ag/AgCl electrodes connected to the compartments via 2 M KCl-agar briges. The data were filtered at 30 Hz and recorded at 2.0 kHz. Current amplitudes were determined by cursor measurements at current increments that indicated insertion of a new channel in the artificial membrane. Single-channel conductance was calculated by dividing the current amplitudes by the applied transmembrane voltage. The histograms of frequency of the insertion events relative to their current amplitudes were constructed. For each histogram, the absolute number of insertion events with certain current amplitude (bin size 2.0 or 5.0 pA) is presented.

For single-channel analysis we used commercial chambers (Warner Instruments) with two compartments (4 ml each) separated by wall with a circular hole (0.05 mm^2^). Both compartments were equipped with magnetic stirrers. As in the case of multiple-channel recordings, the electrode of the *trans* compartment was directly connected to the headstage of a current amplifier. Reported membrane potentials are referred to the *trans* compartment. The capacitance of the bilayer was in the range of 70–110 pF. The data were filtered at 0.4 kHz or 1.0 kHz and recorded at 2.0 kHz. Measurements of reversal potentials were performed by establishing a two-fold (1.0 M KCl *cis*/0.5 M KCl *trans* compartment) salt gradients after formation a stable lipid bilayer. After insertion of a single channel the current was initially measured at 0 mV and than at different membrane potentials.

### Determination of metabolites in blood and urine

#### Uric Acid

Blood was harvested by orbital puncture of anaesthetized mice. Urine was collected in metabolic cages (model 3700M021, Tecniplast) over a time period of 24 h. The samples from male or female mice were analyzed by enzymatic colorimetric tests for uric acid (blood and urine) and creatinine (urine) using the COBAS® Integra diagnostic system (Roche) at the Oulu University Hospital. According to standard clinical practice, the data on urate and allantoin (see below) measurements in urine are given as molar ratios to creatinine. This is a more valuable parameter than molar concentration per volume of urine since urine volume is quite variable between animals.

#### Allantoin

Allantoin was measured in mouse (male) serum and urine as described previously [43] with some modifications. Sera were supplemented with a stable isotope of allantoin ([1-^15^N, 5-^13^C] DL-allantoin, Isotec) as an internal standard and treated with acetone to precipitate proteins. The samples for allantoin detection were prepared by a single step solid-phase extraction of urine and serum using Supelco Discovery DSC-18 column (Supelco). The extracted samples were analyzed on a Polarity™dC18 HPLC column (Waters) interfaced with a Micromass Quattro II mass spectrometer (Micromass). Control urine and serum pools were used for the preparation of calibration standards.

#### Oxalic acid

Urine was collected for 24 h and mice were then administered i.p. 400 mg/kg body mass glycolic acid (Sigma). After injection, urine was collected for two consecutive 24 h time intervals and oxalic acid was determined with an Oxalate kit (Trinity Biotech). The both male and female mice were tested separately.

### Histology and electron microscopy

Liver samples for light microscopy were fixed in 4% (w/v) paraformaldehyde and embedded in paraffin using standard procedures. Sections (5 µm thick) were stained with hematoxylin and eosin. For transmission electron microscopy, samples of liver were fixed in 2.5% (w/v) glutaraldehyde, postfixed in 1% (w/v) osmium-tetroxide, dehydrated in acetone and embedded in Epon Embed 812 (Electron Microscopy Science). Isolated peroxisomes were fixed in 1% (w/v) glutaraldehyde and processed further as described previously [44]. The samples were examined in a Philips EM410 transmission electron microscope.

### Other methods

Composition of subcellular fractions was examined by SDS/PAGE using 15% (w/v) Criterion Precast Gels (Bio-Rad) or home-made 10% (w/v) polyacrylamide gels. Protein bands were visualized by silver or Coomassie blue staining. Immunoblotting was performed using a semi-dry blotter and the blots were incubated with the primary antibodies, followed by detection with alkaline phosphatase-labeled anti-rabbit or anti-goat IgG. Polyclonal antibodies were generated in rabbits against: catalase from bovine liver (Chemicon), rat peroxisomal 3-oxoacyl-CoA thiolase (thiolase), rat sterol carrier protein 2 (SCP-2) (a gift of Dr. K. Wirtz, University of Utrecht, The Netherlands), a synthetic peptide corresponding to a predicted cytosolic domain (amino acids 403–417) of the rat 70 kDa peroxisomal membrane protein (PMP70) sequence (a gift of Dr. S. Alexson, Karolinska Institutet, Stockholm, Sweden), and against murine recombinant Mpv17 protein (ProteinTech). Antibodies against a synthetic peptide corresponding to the N-terminus of mouse PMP22 (NH_2_-APAASRLRVESELG) were prepared by standard procedures. Protein concentration was determined according to Bradford.

### Statistical analysis

Data are presented as means±SD. Significance was determined using a two-tailed Student's ***t*** test. When data from the measurements of blood or urine components with frequent deviations from the normal distribution were analyzed, we used a non-parametric U-test.

## Supporting Information

Figure S1 Pxmp2 disruption strategy and verification of gene inactivation. (A) Schematic representation of the mouse Pxmp2 targeting vector and structure of the locus following gene targeting. The directions of the transcription of Pxmp2 and PoleI are shown by angled arrows. The first exon of PoleI is denoted as a black box and the exons of Pxmp2 are presented as numbered boxes. Relevant restriction sites are shown: S = SacI, E = EcoRI, X = XbaI and B = BamHI. The arrows for lacZ (β-galactosidase gene) and neo (neomycin phosphotransferase gene) indicate the direction of transcription of the corresponding genes. The locations of a 5′ external probe (Probe) for Southern analysis and primers used for PCR genotyping (small arrows) are shown. (B) Southern blot analysis of genomic DNA isolated from livers of wild-type (+/+), heterozygous (+/−) and homozygous (−/−) mice. DNA was digested with SacI and hybridized with the 5′ probe (shown in A). The 5.1 kb and 4.2 kb fragments represent wild-type and targeted alleles, respectively. (C) Northern blot analysis using total RNA isolated from liver and probed with Pxmp2 cDNA.
(1.33 MB TIF)

Figure S2 Peroxisomes from Pxmp2-deficient mouse liver are fragile. (A) Contents of protein and activities of the soluble matrix enzymes in different organelles: peroxisomes (catalase, L-α-hydroxyacid oxidase/HAOX/), lysosomes (acid phosphatase/AP/, β-galactosidase), and mitochondria (glutamate dehydrogenase/GDH/) were measured in the cytosolic fraction and are presented as a percentage of the total amount in postnuclear homogenate from wild-type (dark gray bars) and Pxmp2−/− mice (light gray bars). *P = 0.0001, **P = 0.019 compared with control group, n = 3. Note that only peroxisomal enzymes show an elevated leakage from the particles in Pxmp2−/− mice relative to control (see also Figure S2B). The higher leakage rate of L-α-hydroxyacid oxidase relative to catalase is due to different molecular size of these proteins [1]. (B) Three livers from wild-type (control) and Pxmp2-deficient (Pxmp2−/−) mice, respectively were separately homogenized in isolation medium containing 0.25 M sucrose as an osmoprotectant. The nuclei were sedimented and the postnuclear homogenates were centrifuged at 100,000 gmax for 60 min to obtain the cytosolic fraction. Samples from cytosol (marked as c) and homogenate (h) were used for immunodetection of peroxisomal proteins: 3-oxoacyl-CoA thiolase (thiolase), sterol carrier protein 2 (SCP2, this protein shows dual, peroxisomal/cytoplasmic, localization in the liver of rodents, [2]), sterol carrier protein 2/3-oxoacyl-CoA thiolase (SCP2/Thiolase), peroxisomal membrane protein 70 (PMP70), and Pxmp2. The peroxisomal membrane proteins PMP70 and Pxmp2 were not detected in the cytosolic fraction (data not shown) indicating that only soluble matrix proteins leaked out of the particles during homogenization. Note an increased leakage of matrix proteins from Pxmp2-deficient peroxisomes. (C) Immunodetection of peroxisomal 3-oxoacyl-CoA thiolase (Thiolase) and peroxisomal membrane protein 70 (PMP70) in fractions obtained after Nycodenz density gradient centrifugation of the postnuclear homogenates prepared from livers of wild-type (upper bands) and Pxmp2−/− (lower bands) mice. Proteins from equal volumes of each fraction of the gradient were separated by SDS-PAGE and immunoblotted. Note that the peroxisomal membrane marker PMP70 is found only in the bottom gradient fractions, indicating that the wild-type and Pxmp2-deficient peroxisomes enter the gradient. In contrast, the soluble matrix protein thiolase shows a dual distribution with a significant part of the enzyme detected in the top fractions containing mainly cytosolic proteins. It is noteworthy that, like L-α-hydroxyacid oxidase (see Figure 1C), thiolase shows more extensive leakage from Pxmp2-deficient peroxisomes than from wild-type control. (D) Distribution of protein and marker enzymes for different organelles: peroxisomes (urate oxidase), microsomes (esterase), mitochondria (GDH), and lysosomes (AP), and activity of a marker for cytosol (fructose phosphate isomerase/FPI/) in fractions obtained after Nycodenz gradient centrifugation of postnuclear homogenates from wild-type (dark gray bars) and Pxmp−/− (light gray bars) mouse livers. The total amounts of protein and enzymes activity loaded on the gradients were: protein 150 mg and 166 mg for wild-type and Pxmp2-deficient samples respectively; esterase 55.2 U and 57.0 U; AP 7.2 U and 7.4 U; urate oxidase 2.4 U and 2.5 U; GDH 11.6 U and 11.0 U, and FPI 39.0 U and 42.4 U. The yield of a total protein in the fractions enriched with peroxisomes (fractions 2–5) was 2.10±0.05% (wild-type) and 1.64±0.04% (Pxmp2−/−, P = 0.044, n = 3) relative to a protein amount loaded on the gradients. Note that the localization of the peroxisomal nucleoid marker urate oxidase near the bottom of the gradient is similar to the distribution of PMP70 (marker for peroxisomal membrane, see Figure S2C). The results agree with data from electron microscopy (see Figure 1B and Figure S2E) showing the presence in the bottom gradient fractions of near intact peroxisomes side by side with particles that contain membrane and nucleoid but are poor in matrix proteins (peroxisomal “ghosts”). The gradient distribution of PMP70 indicates that the Pxmp2-deficient samples contain more peroxisomal “ghosts” (which have lower equilibrium density relative to intact peroxisomes [1]) than wild-type samples. As seen from the figure, the Pxmp2 deficiency does not influence the distribution in the gradient of subcellular organelles other than peroxisomes, such as mitochondria, lysosomes and fragments of endoplasmic reticulum (microsomes). (E) Electron micrograph of peroxisomes purified from livers of wild-type (panels 1 and 2) and Pxmp2−/− (panel 3) mice. Original magnification, ×8400; bar, 500 nm (panels 1 and 3) or ×15000; bar 200 nm (panel 2), respectively. The electron density of peroxisomes roughly reflects the amount of soluble matrix proteins inside the organelle [1], [3]. The particles with high density, visible in preparations from wild-type mice (panels 1 and 2, see also Figure 1B), represent near intact peroxisomes filled with matrix proteins and surrounded by a single membrane (marked by an arrow on panel 2). The same preparations also contain peroxisomes with intermediate electron density (marked by asterisks on panel 1) as well as particles with low electron density (marked by arrow on panel 1) showing that some peroxisomes have been partially disrupted during isolation. The poor electron density of most of the particles in the peroxisomal fraction isolated from livers of Pxmp2−/− mice (panel 3, see also Figure 1B) indicates leakage of soluble matrix proteins from the organelles, leading to formation of peroxisomal “ghosts.” (F) Effect of PEG1500 in the isolation medium on the leakage of enzymes from different organelles. The activity of the enzymes and protein content were measured in the cytosolic fraction and are presented as a percentage of the total amount in postnuclear homogenate which has been obtained using isolation medium containing 0.25 M sucrose (dark gray bars) or 0.16 M sucrose and 12% (w/v) PEG1500 (light gray bars) as osmoprotectants (see legend to Figure S2B and ref. [1] for more details). HAOX: *P = 0.006, **P = 0.00012; Catalase: *P = 0.0005, **P = 0.007. Note that PEG1500 decreases the leakage of the soluble matrix enzymes (HAOX, catalase) not only from wild-type peroxisomes but also from the Pxmp2-deficient particles. Moreover, in contrast to higher fragility of the Pxmp2-deficient peroxisomes relative to wild-type control when 0.25 M sucrose has been used as an osmoprotectant, the particles show the same level of fragility in the presence of PEG1500. However, the latency of urate oxidase was similar in peroxisomal preparations isolated with or without PEG1500 as an osmoprotectant in both groups, wild-type and Pxmp2-deficient mice. (G) Activity of L-α-hydroxyacid oxidase in fractions obtained after Nycodenz gradient centrifugation of the postnuclear homogenates prepared from livers of Pxmp2-deficient mice. The homogenization medium contained 0.25 M sucrose (light gray bars) or 0.16 M sucrose and 12% (w/v) PEG1500 (dark gray bars). Recoveries of the enzyme were 110% (sucrose) and 108% (PEG1500), respectively. See legend to Figure 1C for more details. Note decrease in the leakage of HAOX from peroxisomes when homogenates were prepared using PEG1500 as an osmoprotectant. (H) Immunodetection of peroxisomal thiolase in fractions obtained after Nycodenz gradient centrifugation of liver homogenates from Pxmp2−/− mice. The homogenates were prepared in the presence of 0.25 M sucrose (upper band) or 0.16 M sucrose and 12% (w/v) PEG1500 (lower band) as osmoprotectants. See legend to Figure S2C for more details. (I,J) Illustration explaining an osmotic behavior of peroxisomes during isolation. I, Homogenization using an isolation medium containing an inappropriate osmoprotectant (e.g., sucrose is a very poor osmoprotectant for mammalian peroxisomes because it easily penetrates the membrane [1], [4]) leads to an abrupt dropin the concentration of solutes outside peroxisomes, provoking a flow of water into the particles and an outflow of solutes, the movement of water occurs by spontaneous diffusion across the phospholipids bilayer as well as through the channels; J-1, movement of water (small black circles) and solutes (large gray circles) in wild-type peroxisomes containing at least two types of channels, marked on the picture with single and double walls, respectively; J-2, The same picture as before, but one type of channels (with double walls) is missing. Note that outflow of solutes is restricted; J-3 Presence of PEG1500 prevents water to flow into the particles decreasing an osmotic pressure in peroxisomes (see Text S2).
(0.92 MB TIF)

Figure S3 Permeability of Pxmp2-deficient peroxisomal membrane to substrates of peroxisomal enzymes *in vitro* and *in vivo*. (A) Effect of Pxmp2 deletion on the latency of peroxisomal enzymes: L-α-hydroxyacid oxidase (HAOX), catalase, lactate dehydrogenase (LDH), and glycerol-3-phosphate dehydrogenase (GPDH). The last two enzymes are NADH-dependent. “Free” activity of the enzymes in peroxisomes isolated from wild-type (dark gray columns) and Pxmp2−/− (light gray columns) mouse livers is presented as a percentage of the “total” activity, *P = 0.0001, n = 7–8. Note that the “free” activity of HAOX is significantly lower in Pxmp2-deficient peroxisomes than in wild-type control (see Text S3 for details). (B) Illustration explaining the mechanism of peroxisomal enzyme latency. Substrate (dark square) penetrating the membrane through the channel with velocity V1 is converted by the peroxisomal enzyme into the corresponding product (dark circle) with velocity V2. (C) Dependence of urate oxidase activity on substrate concentration. Activity of urate oxidase was measured in a purified peroxisomal fraction treated with 0.05% (v/v) Triton X-100 to recover “total” activity of the enzyme. The concentration of uric acid was detected spectrophotometrically using the molar extinction coefficient ε292 nm = 7.6×103 M-1 cm-1. Arrows 2 and 3 indicate “free” activity of urate oxidase in wild-type and Pxmp2-deficient peroxisomes, respectively relative to the “total” activity that was detected at the final substrate concentration of 150 µM (see Text S3 for details). (D) A Lineweaver-Burk plot was calculated using data points from Figure S3C. The results indicate that the dependence of urate oxidase activity on substrate concentration follows Michaelis-Menten kinetics. (E) Samples of postnuclear homogenates were subjected to SDS-PAGE, electroblotted and stained with anti-urate oxidase antibodies (Santa-Cruz biotechnology). Note that the content of urate oxidase protein is similar in wild-type and Pxmp2-deficient mice. (F) Detection of oxalic acid in urine. Oxalic acid is the final product of glycolic acid oxidation. The metabolism of glycolic acid in mammals proceeds in cytoplasm with formation of oxalic acid and in peroxisomes with production of glycine [5]. One can expect that restriction in access of glycolic acid to peroxisomes might cause elevated oxidation of this compound in cytoplasm, resulting in an increase in the production of oxalic acid. Wild-type (dark circle) and Pxmp2−/− (open circle) mice were injected with glycolic acid and excretion of oxalic acid in urine was measured. The absolute quantity of excreted oxalic acid was calculated as µmoles in the total urine collected within 24 h per 100 g body mass (left panel). The urine was collected for 24 h before glycolate injection (1), for 24 h after injection (2) and from 24 h to 48 h after injection (3). The difference between the content of oxalic acid excreted in urine collected 24 h before and 24 h after the injection of glycolic acid is shown in the right panel. The difference was calculated for each mouse. *P = 0.035. The data for female mice are shown. Note that the levels of oxalic acid excreted before injection of glycolic acid were similar in both groups of mice. However, after injection of glycolic acid, a higher rate of oxalic acid excretion in Pmpx2-deficient mice, as compare to wild-type control, was detected. Measurements of the “total” activity of enzymes participating in oxidation of glycolic acid in peroxisomes (HAOX, alanine-glyoxylate aminotransferase) or in cytoplasm (LDH) in liver homogenates did not reveal any difference between Pxmp2−/− and wild-type mice, respectively (data not shown).
(1.86 MB TIF)

Figure S4 Figure S4. Detection of channel-forming activity in insect cells. (A) Isolation of a Pxmp2-enriched membrane fraction from insect cells (see Methods for details). According to marker enzyme activity detection (see Figure S4B), the postmitochondrial particle fraction (PPF) was enriched with fragments of endoplasmic reticulum (microsomes) and (micro)peroxisomes. Samples from each fraction containing an equal amount of protein (40 µg) were subjected to SDS/PAGE and immunoblotted with antibodies against mouse Pxmp2. Control: mock transfected cells. (B) Characterization of subcellular fractions isolated from insect cells. Catalase, GDH, esterase, AP, and LDH were used as markers for (micro)peroxisomes, mitochondria, endoplasmic reticulum, lysosomes, and cytosol, respectively. Specific activities of the enzymes were measured in the corresponding fractions isolated from mock-transfected (dark gray columns) and Pxmp2-transfected (light gray columns) insect cells and are presented as percentage of activity in post-nuclear homogenates (100%). Note that in addition to the marker for endoplasmic reticulum (esterase), the postmitochondrial particle fraction (PPF) shows a high catalase activity, implying that this fraction contains a significant amount of (micro)peroxisomes. The PPF also contains the highest concentration of recombinant Pxmp2 (see Figure S4A) and was used for detection of the pore-forming activity (see Figure 2C and 2D). (C) Effect of anti-Pxmp2 antibody on the channel-forming activity. The PPF was isolated from insect cells expressing Pxmp2, and an aliquot of solubilized protein (400 µg) was incubated (30 min at +4°C) with IgG (5 µg) purified from pre-immune (left panel) or immune (right panel) serum. The preparations were then used for multiple-channel recording. Note the decrease in the amount of insertion events with current amplitudes 20–35 pA (1.0 M KCl, +20 mV) after treatment of the samples with anti-Pxmp2 antibodies. The total number of insertion events: left panel 251, right panel 295.
(1.40 MB TIF)

Figure S5 Figure S5. Purification and characterization of the quaternary structure of Pxmp2. (A) Partial purification of Pxmp2 (see: Text S1). Proteins were separated by SDS-PAGE and stained with silver nitrate. Lane 1, isolated peroxisomes (10 µg); lane 2, peroxisomal membrane preparation after treatment at pH 11.3 (8 µg); lane 3, peroxisomal membranes solubilized by Genapol (5 µg); lane 4, fraction enriched with Pxmp2 obtained after ion-exchange chromatography and first size-exclusion chromatography steps (0.6 µg). The protein identified by MALDI-TOF mass spectrometry as Pxmp2 is marked with an asterisk. The migration of molecular mass (in kDa) markers (LMW Calibration kit, Bio-Rad) is indicated at the left side. (B) Size-exclusion chromatography of partially purified Pxmp2 before (upper panel) and after (lower panel) treatment of the protein with n-dodecyl β-D-maltoside (DDM, see: Text S1). Proteins in 200 µl column fractions were separated by SDS-PAGE, transferred to nitrocellulose, and incubated with anti-Pxmp2 antibodies. (C) Mobility of Pxmp2 on a SuperdexTM200 column. The calibration curve was generated by plotting the log of the molecular mass of the standards versus mobility on the column. The molecular mass markers are: (1) catalase, 240 kDa; (2) lactate dehydrogenase, 140 kDa; (3) bovine serum albumin, 66 kDa; (4) carbonic anhydrase, 29 kDa; and (5) cytochrome c, 12.4 kDa. The void volume was determined using Blue Dextran (2000 kDa), which was detected in fractions 1–3. The mobility of the different forms of Pxmp2 are marked by arrows: (I) Pxmp2 partially purified in the presence of Genapol (see Figure S5B, upper panel); (II) partially purified Pxmp2 treated with DDM (see Figure S5B, lower panel); (III) Pxmp2 after thermal treatment (see Figure 5D, lower panel) or after solubilization from the membrane using 2.0% (w/v) SDS (blot not shown). (D) Mobility of purified Pxmp2 on a SuperdexTM200 column before (upper panel) and after (lower panel) treatment of the protein at 75°C for 6 min. Traces of Pxmp2 visible in fraction 36 are apparently caused by retention of some molecules of denaturized protein on the column. (E) Cross-linking and immunoblot analysis of purified Pxmp2. The protein eluted from a SuperdexTM200 column was treated with ethylene glycolbis(sulfosuccinimidylsuccinate) (Sulfo-EGS, right lane). Control sample did not have cross-linker (left lane). The immunoreactive product of the cross-linking is marked by an asterisk. (F) Cross-linking of the His-tagged Pxmp2 protein. The expression of the His-tagged Pxmp2 was carried out using the BAC-TO-BACTM Baculovirus Expression System (Invitrogen) and Sf9 insect cell line (A. Isomursu, unpublished data). The postmitochondrial particles fraction (PPF) was isolated and the proteins were solubilized with 0.5% (v/v) Genapol X-080. The aliquots of the solubilized proteins (about 50 µg) were treated with disuccinimidyl suberate (DSS, Pierce) for 30 min at room temperature. DSS concentrations were: 0.1, 0.2, 0.5, 1.0 mM (lanes 2,3,4,5). Control sample was without cross-linker (lane 1). After cross-linking, the proteins were separated by SDS-PAGE followed by immunoblot analysis using anti-His-Tag antibodies (Novagen). It appears that application of these antibodies led to more valid results if compare with the anti-Pxmp2 antibodies which produced the only low signal with the cross-linked protein. Note that, similar to purified Pxmp2 (see Figure S5E), the cross-linking of the recombinant protein leads to appearance of molecules with the size (about 80 kDa) three times larger than the size of the His-tagged Pxmp2 protein (about 27 kDa). It is highly unlikely that the mouse recombinant Pxmp2 produces heteromers with the insect cell proteins. Therefore, the data support the notion that the Pxmp2 protein forms a homotrimer in the membrane.
(1.32 MB TIF)

Figure S6 Electrophysiological analysis of purified Pxmp2. (A) Channel conductance as a function of KCl concentration. The figure presents measurements (multiple channel recording) of the increments in current which show peaks in channel activity at an average conductance of 1.3 nS (upper line) or 0.45 nS (lower line) in 1.0 M KCl (see Figure 3B and 3C). The mean conductance ±SD of 30–40 single events is shown. (B) Current traces of a bilayer containing one Pxmp2 pore-forming protein (the insertion event is visible on the upper trace) under asymmetric salt conditions: 1.0 M KCl cis/0.5 M KCl trans compartment. The holding potentials are marked. The data were filtered at 1.0 kHz and recorded at 2.0 kHz. (C) Current-voltage relationship of the Pxmp2 pore-forming protein under asymmetric salt concentrations; data points are averages of at least 6 independent measurements±S.D.
(1.37 MB TIF)

Figure S7 Determination of the size of the Pxmp2 channel. (A) Average conductance of high (1.3 nS in 1.0 M KCl, light gray bars) and low (0.45 nS in 1.0 M KCl, open bars) conductance Pxmp2 channels in different salt solutions (1.0 M, final concentration). The average conductance±SD (G, right ordinate axis) was calculated from at least 40 single events. Bulk electrolyte conductivity (sigma, left ordinate axis) of the corresponding salt solutions is shown as dark-gray bars. (B) Estimation of the size of the Pxmp2 channel using cations of different hydrated radii. The relative conductance rates (G/sigma) for high- (dark circle) and low- (open circle) conductance channels (see Figure S7A) were normalized to those of Rb+ (100 arbitrary units) and logarithms of the resulting values were plotted against the hydrated radii of the corresponding cations: Rb, 0.105 nm; KCl, 0.111 nm; NaCl, 0.163 nm; LiCl, 0.216 nm; Tris-Cl, 0.321 nm; TEA-Cl, 0.426 nm. The hydrated radii of cations [6] and non-electrolytes [7] (see below) including polyethylene glycols (PEGs, Sigma) were taken from the corresponding publications. (C) Estimation of the size of the Pxmp2 channel (high-conductance state) using polymer exclusion method. Left panel, the average conductance was calculated from 40–60 single events. Ratios of the channel conductance without (Go) and with (G) non-electrolyte was plotted against hydrated radii of non-electrolytes: ethylene glycol, 0.26 nm; glycerol, 0.31 nm; arabinose, 0.34 nm; PEG200, 0.43 nm; PEG300, 0.60 nm; PEG400, 0.70 nm; PEG600, 0.78 nm; PEG1000, 0.94 nm; PEG2000, 1.22 nm; PEG3400, 1.63 nm. The 20% (w/v) solutions of non-electrolytes containing 1.0 M KCl (final concentration) were added to both halves of the chamber. Right panel: second derivative of the data from left panel showing two turning points. The upper one indicates the size of non-electrolytes which partition into the pore become restricted. The lower one indicates the maximal size of non-electrolytes which still able to penetrate the channel (see Text S1 for details). (D) Structure of “bulky” (ATP, NADH) and small (uric acid, glycine) solutes.
(0.31 MB TIF)

Text S1 Supporting methods.
(0.04 MB DOC)

Text S2 Fragility of Pxmp2-deficient peroxisomes *in vitro*.
(0.03 MB DOC)

Text S3 Latency of peroxisomal enzymes.
(0.03 MB DOC)
