# Supplementary figures and images for: Pxmp2 Is a Channel-Forming Protein in Mammalian Peroxisomal Membrane
Source: PLoS One. 2009 Apr 7;4(4):e5090. doi: 10.1371/journal.pone.0005090 (PMC2662417; doi:10.1371/journal.pone.0005090)

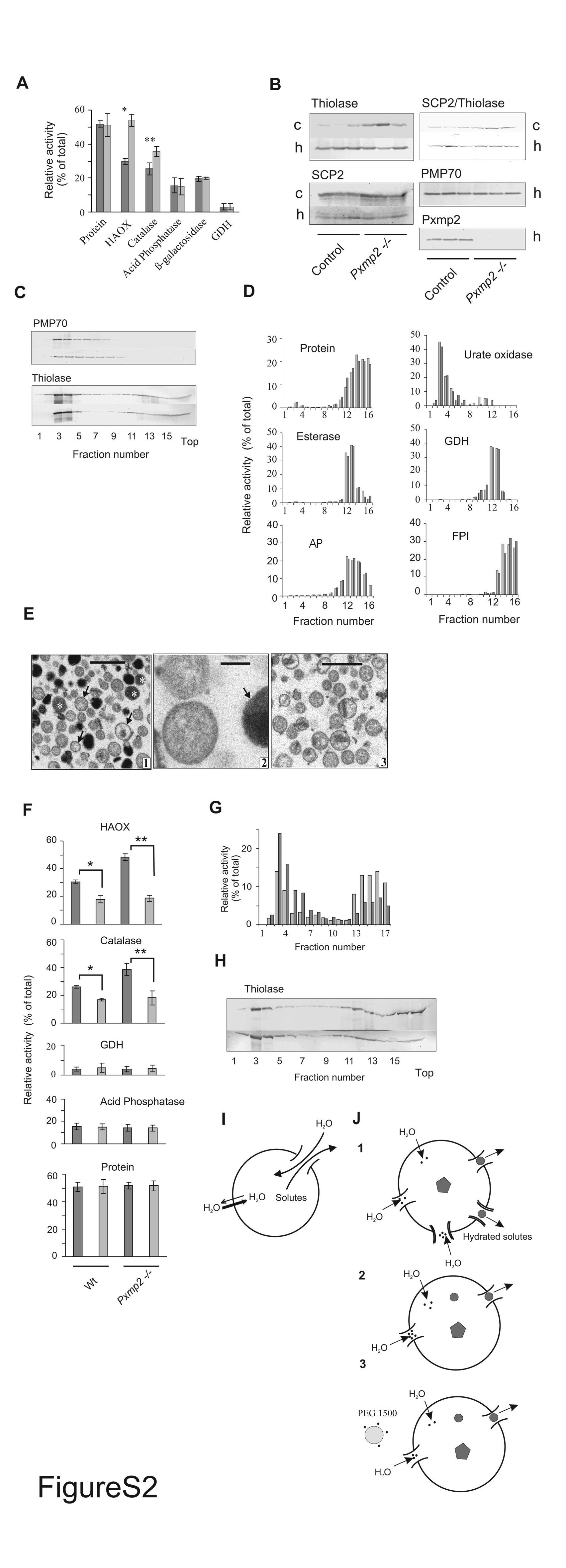

Supplement: Figure S2 — Peroxisomes from Pxmp2-deficient mouse liver are fragile. (A) Contents of protein and activities of the soluble matrix enzymes in different organelles: peroxisomes (catalase, L-α-hydroxyacid oxidase/HAOX/), lysosomes (acid phosphatase/AP/, β-galactosidase), and mitochondria (glutamate dehydrogenase/GDH/) were measured in the cytosolic fraction and are presented as a percentage of the total amount in postnuclear homogenate from wild-type (dark gray bars) and Pxmp2−/− mice (light gray bars). *P = 0.0001, **P = 0.019 compared with control group, n = 3. Note that only peroxisomal enzymes show an elevated leakage from the particles in Pxmp2−/− mice relative to control (see also Figure S2B). The higher leakage rate of L-α-hydroxyacid oxidase relative to catalase is due to different molecular size of these proteins [1]. (B) Three livers from wild-type (control) and Pxmp2-deficient (Pxmp2−/−) mice, respectively were separately homogenized in isolation medium containing 0.25 M sucrose as an osmoprotectant. The nuclei were sedimented and the postnuclear homogenates were centrifuged at 100,000 gmax for 60 min to obtain the cytosolic fraction. Samples from cytosol (marked as c) and homogenate (h) were used for immunodetection of peroxisomal proteins: 3-oxoacyl-CoA thiolase (thiolase), sterol carrier protein 2 (SCP2, this protein shows dual, peroxisomal/cytoplasmic, localization in the liver of rodents, [2]), sterol carrier protein 2/3-oxoacyl-CoA thiolase (SCP2/Thiolase), peroxisomal membrane protein 70 (PMP70), and Pxmp2. The peroxisomal membrane proteins PMP70 and Pxmp2 were not detected in the cytosolic fraction (data not shown) indicating that only soluble matrix proteins leaked out of the particles during homogenization. Note an increased leakage of matrix proteins from Pxmp2-deficient peroxisomes. (C) Immunodetection of peroxisomal 3-oxoacyl-CoA thiolase (Thiolase) and peroxisomal membrane protein 70 (PMP70) in fractions obtained after Nycodenz density gradient centrifuga [file pone.0005090.s002.tif]

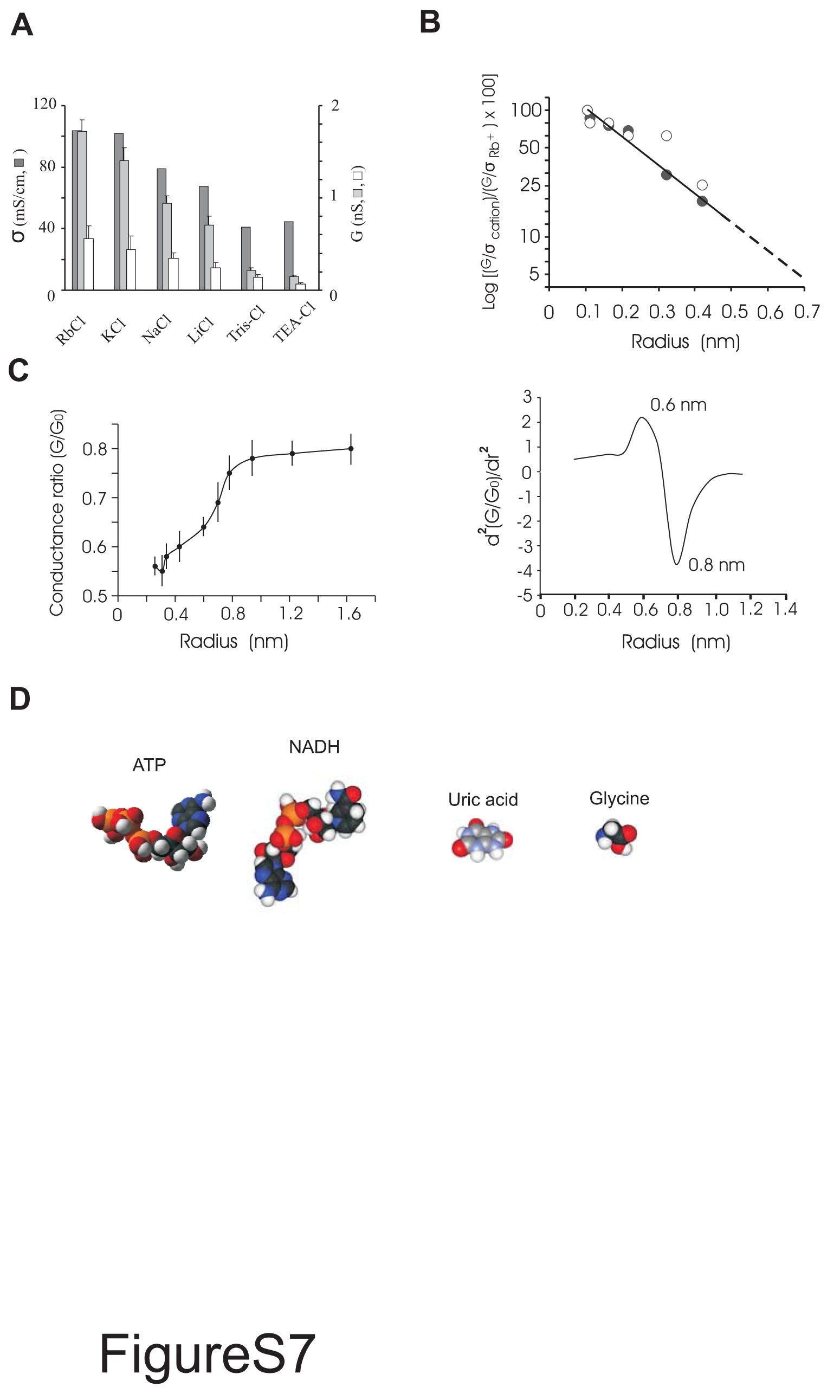

Supplement: Figure S7 — Determination of the size of the Pxmp2 channel. (A) Average conductance of high (1.3 nS in 1.0 M KCl, light gray bars) and low (0.45 nS in 1.0 M KCl, open bars) conductance Pxmp2 channels in different salt solutions (1.0 M, final concentration). The average conductance±SD (G, right ordinate axis) was calculated from at least 40 single events. Bulk electrolyte conductivity (sigma, left ordinate axis) of the corresponding salt solutions is shown as dark-gray bars. (B) Estimation of the size of the Pxmp2 channel using cations of different hydrated radii. The relative conductance rates (G/sigma) for high- (dark circle) and low- (open circle) conductance channels (see Figure S7A) were normalized to those of Rb+ (100 arbitrary units) and logarithms of the resulting values were plotted against the hydrated radii of the corresponding cations: Rb, 0.105 nm; KCl, 0.111 nm; NaCl, 0.163 nm; LiCl, 0.216 nm; Tris-Cl, 0.321 nm; TEA-Cl, 0.426 nm. The hydrated radii of cations [6] and non-electrolytes [7] (see below) including polyethylene glycols (PEGs, Sigma) were taken from the corresponding publications. (C) Estimation of the size of the Pxmp2 channel (high-conductance state) using polymer exclusion method. Left panel, the average conductance was calculated from 40–60 single events. Ratios of the channel conductance without (Go) and with (G) non-electrolyte was plotted against hydrated radii of non-electrolytes: ethylene glycol, 0.26 nm; glycerol, 0.31 nm; arabinose, 0.34 nm; PEG200, 0.43 nm; PEG300, 0.60 nm; PEG400, 0.70 nm; PEG600, 0.78 nm; PEG1000, 0.94 nm; PEG2000, 1.22 nm; PEG3400, 1.63 nm. The 20% (w/v) solutions of non-electrolytes containing 1.0 M KCl (final concentration) were added to both halves of the chamber. Right panel: second derivative of the data from left panel showing two turning points. The upper one indicates the size of non-electrolytes which partition into the pore become restricted. The lower one indicates the maximal size of non-electrolytes which still [file pone.0005090.s007.tif]
