# Supplementary material for: Pxmp2 Is a Channel-Forming Protein in Mammalian Peroxisomal Membrane
Source: PLoS One. 2009 Apr 7;4(4):e5090. doi: 10.1371/journal.pone.0005090 (PMC2662417; doi:10.1371/journal.pone.0005090)
Supplement: Text S1 — Supporting methods. (0.04 MB DOC) [file pone.0005090.s008.doc]

**Supporting methods S1**

**Subcellular fractionation and isolation of mouse liver peroxisomes**

For measurements of enzyme activity and Western blot analysis, liver tissue collected from male 6-month-old mice after an overnight fast was homogenized in 0.25 M sucrose, 20 mM MOPS, pH 7.4, 1 mM EDTA. For a comparative study of peroxisomes from *Pxmp2*^-/-^ and wild-type mice, purification of the particles was performed at the same time for both groups of animals (8-10 mice in each group per isolation experiment). For morphological examination of peroxisomes and for detection of the latency of peroxisomal enzymes, the particles were isolated using Nycodenz gradient centrifugation as described previously [1] but without a Percoll gradient centrifugation step. The Nycodenz gradients were formed using 3 ml cushions with density 1.25 g/cm^3^.

To obtain highly purified peroxisomes for use in the reconstitution assay in lipid bilayer, we exploited the procedure described previously [1,8] with some modifications: to further increase purity of the peroxisomes, Optiprep gradient centrifugation was carried out in 60 ml centrifugation tubes using a fixed-angle rotor. After centrifugation in Optiprep gradients, the gradient fractions most enriched with peroxisomes (detected by measuring activity of the marker enzyme L-α-hydroxyacid oxidase) were collected and diluted 4-5 times with the isolation medium. The organelles were then sedimented by centrifugation at 100,000 g_max_ for 45 min, resuspended in 10 mM MOPS, pH 7.2 and stored in aliquots at -80^o^C before use. For purification of native Pxmp2, mice were maintained for 2 weeks on a standard diet containing 0.3% (v/w) clofibrate. Isolation of peroxisomes was performed as described above, although the Percoll gradient centrifugation step was omitted. The purified particles were diluted with 20 mM MOPS, pH 7.2 to 2.0-2.5 mg/ml, sonicated (6 cycles, 15 s each, amplitude 15 μm) and sedimented at 120 000 g_max_ for 45 min. A pellet containing peroxisomes depleted in matrix proteins (peroxisomal ‘ghosts’) was resuspended in 1.0 M KCl, 20 mM Tris-Cl, pH 9.8, sonicated and sedimented as before. The resulting peroxisomal preparation was suspended in 0.1 M Na_2_CO_3_, pH 11.3 and rotated for 2h at 4^o^C to remove peripheral membrane proteins. The peroxisomal membranes were collected by centrifugation at 200 000 g_max_ for 45 min and the integral membrane proteins were solubilized by overnight incubation of the membrane preparation in 20 mM MOPS, pH 7.2, containing 5% (v/v, final concentration) Genapol X-080 at 4^o^C. The samples were then used for isolation of native Pxmp2 protein.

**Measurement of enzyme activities and latency determination**

Activities of marker enzymes for subcellular organelles: mitochondria (glutamate dehydrogenase [9]), lysosomes (acid phosphatase [10] and β-galactosidase [11], endoplasmic reticulum (esterase [12]), and peroxisomes (L-α-hydroxyacid oxidase [13] and catalase [14]) were measured according to standard procedures. Activity of urate oxidase was measured by direct registration (292 nm) at pH 8.2 of the disappearance of urate (latency determination [9]) or by a coupled assay described elsewhere (detection of the activity in gradient fractions, [10]). Activities of fructose phosphate isomerase [15] and glycerol-3-phosphate dehydrogenase [16] were measured with fructose-6-phosphate and dihydroxyacetone phosphate as substrates, respectively. Alanine glyoxylate aminotransferase activity was measured by detection of pyruvate formation after trapping glyoxylate by Tris-Cl buffer [17]. Units of enzyme activity are given as μmol of substrate consumed or product formed per minute. Catalase, which shows first-order reaction kinetic, is expressed in units defined previously [9].

The latency of the enzymes was detected in freshly prepared peroxisomal fractions after resedimentation of the particles as described [4]. ‘Free’ enzyme activity was measured before disruption of the peroxisomal membrane by Triton X-100 (0.05%, w/v, final concentration) which was used to reveal ‘total’ enzyme activity.

**Purification and characterization of the quaternary structure of native Pxmp2 from mouse liver peroxisomes**

Genapol-solubilized peroxisomal membrane proteins (12-14 mg) were separated from Genapol-insoluble material by centrifugation and applied onto a cassette of ion-exchange cartridges consisting of 1 Mono-Q Econo-Pac cartridge connected in head to tail configuration to 2 Mono-S cartridges (5 ml each, Bio-Rad). The cassette was equilibrated with 20 mM MOPS, pH 7.2, 0.2% (v/v) Genapol. After washing with the equilibration buffer (35 ml), the Mono-Q cartridge was removed and the Mono-S cartridges were washed once more with the same buffer containing 0.4 M NaCl (35 ml). The bound proteins were eluted with a 50 ml 0.4 – 2.0 M linear NaCl gradient at a flow rate of 1.4 ml/min. Fractions of 2.0 ml were collected and analyzed for Pxmp2 by dot blot (3μl of each fraction). Immunoreactive fractions were further analyzed by Western blot and by SDS-PAGE followed by silver-staining. Pxmp2 usually eluted as a broad band (about 20 fractions) between 0.9 and 2.0 M NaCl. The initial 8-10 fractions containing Pxmp2 were pooled, concentrated on Amicon membranes with a cut off of 10 kDa (Millipore) and applied onto a Superdex^TM^ 200 column (Pharmacia) equilibrated with 20 mM MOPS, pH 7.2, 0.2% (v/v) Genapol, and 0.15 M NaCl. The protein sample was chromatographed at a flow rate of 0.8 ml/min and fractions of 2.0 ml were collected. Pxmp2 eluted just after the void volume of the column. Two to three immunoreactive fractions were pooled, concentrated and treated with 20 mM MOPS, pH 7.2 containing 4% (w/v) n-dodecyl β-D-maltoside (DDM, Sigma) overnight at 4^o^C. The sample was then chromatographed once more on a Superdex^TM^200 column equilibrated with 20 mM MOPS, pH 7.2, 0.1% (w/v) DDM, and 0.15 M NaCl. Treatment with DDM leads to a marked decrease in mobility of Pxmp2 on the column (see below). The immunoreactive fractions were collected, concentrated, and stored on ice for detection of the pore-forming activity.

The purity of Pxmp2 was analyzed by SDS-PAGE followed by silver staining or immunoblotting. To confirm the authenticity of Pxmp2, the protein band was excised from a gel stained with Coomassie Blue, digested in-gel with trypsin and identified from the peptide mass fingerprint by matrix-assisted laser desorption-ionization mass spectrometry (MALDI-MS).

A Superdex^TM^200 column connected to an ÄKTA-prime (Amersham) system and calibrated with protein standards (Sigma) was used to determine the molecular mass of native Pxmp2. Chemical cross-linking experiments using purified Pxmp2 were performed at room temperature for 20 min with 0.2 mM ethylene glycolbis(sulfosuccinimidylsuccinate) (Sulfo-EGS, Pierce).

Mouse Pxmp2 is less abundant than the protein from rat liver peroxisomes [18] and is hardly detectable in the total peroxisomal fraction or in preparations of peroxisomal membranes in silver-stained SDS-PAGE (see Fig. S5A, lines 1-3). However, the protein constitutes a predominant component of the fraction obtained after ion-exchange and size-exclusion chromatographies (lane 4). Based on size-exclusion chromatography, the predicted size of native Pxmp2 solubilized by Genapol is 200 kDa (Fig. S5B, upper panel and Fig. S5C). However, the use of other detergents, such as DDM leads to dissociation of the 200 kDa form and results in the appearance of a Pxmp2 form with an estimated molecular mass of 66 kDa (Fig. S5B, lower panel and Fig. S5C). We exploited this special feature of Pxmp2 in our purification procedure. Treatment of partially purified Pxmp2 obtained after size-exclusion chromatography in the presence of Genapol (see above) by DDM followed by repetition of the size-exclusion chromatography led to almost complete purification of the protein (Fig. 3A, left panel). The identity of the isolated Pxmp2 was confirmed by Western blotting (right panel) and mass spectrometric analysis.

To verify the molecular mass of isolated Pxmp2, the protein was subjected to thermal treatment followed by size-exclusion chromatography. As expected, two forms of the protein were detected, with estimated molecular masses of 66 kDa and 22 kDa, respectively (Fig. S5D), indicating that Pxmp2 is a homotrimer. This was confirmed by cross-linking experiments (Fig. S5E,F) and by using SDS to solubilize Pxmp2 from peroxisomal membranes. As with the situation for the thermal treatment, incubation of Pxmp2 with 0.4% (w/v) SDS resulted in two immunoreactive bands after size-exclusion chromatography, with predicted molecular masses of 66 kDa and 22 kDa, respectively (data not shown).

**Estimation of the pore size of the Pxmp2 channel**

We performed additional reconstitution assays in lipid bilayers with salts other than KCl in order to obtain information on the pore size of the channels formed by Pxmp2 (see: Fig. S7A,B). The conductance sequence of the different salts for high and low conductance channels (1.3 nS and 0.45 nS in 1.0 M KCl, respectively) was RbCl > KCl > NaCl > LiCl > TrisCl > Tetraethylammonium chloride (TEACl), indicating that the single-channel conductance followed the mobility sequence of the cations in the aqueous phase. The poor conductance for the large cations (Tris^+^ and TEA^+^) suggested that the rate of their penetration is limited by the size of the pore. This was confirmed when the relative conductance rates (G/σ) for different cations, given on a logarithmic scale were plotted against the hydrated radii of these cations (Fig. S7B). The curve obtained shows linear dependence between the plotted parameters, and predicts that the rates of penetration for cations with hydrated radii between 0.7-0.8 nm are only few percentage points of that for Rb^+^, indicating that these dimensions are appropriate values for the channel size. Importantly, the specific conductance rates (G/σ) of the cations for the low conductance channel (0.45 nS in 1.0 M KCl) were comparable with the levels obtained for the high conductance channel (1.3 nS in 1.0 M KCl), implying that both types of channels have a similar size.

To obtain more information about channel size, we measured the channel conductance in the presence of highly concentrated solutions of non-electrolytes with different hydrated radii (Fig. S7C). The detailed theoretical background of the method is given elsewhere [7,19,20]. Briefly, high concentrations of non-electrolytes increase the viscosity of aqueous solutions and thus decrease the mobility of ions. The conductance of a channel will decrease when the non-electrolytes are small enough to enter the pore and increase the viscosity there. When non-electrolytes are too big to enter the pore, the conductance of a channel is close to that in the absence of the non-electrolytes. The lowest conductance rates were observed for small molecules: ethylene glycol, glycerol, and arabinose, indicating that these compounds easily enter the Pxmp2 channel (Fig. S7C). A gradual increase in conductance in the presence of PEGs 200–400 reflects growing restriction in the penetration of the corresponding molecules into the channel. The dimensions of the narrowest space of the channel (channel friction) could correspond to the size of the smallest impermeable non-electrolyte. The channel conductance reached a plateau when PEG600 was tested, indicating that this compound might be prevented from entering into the narrowest space of the pore. Its hydrated radius, 0.78 nm, is in a good agreement with the channel radius of 0.7-0.8 nm that was predicted from the cation permeability measurements (see above).
