# Supplementary material for: Pxmp2 Is a Channel-Forming Protein in Mammalian Peroxisomal Membrane
Source: PLoS One. 2009 Apr 7;4(4):e5090. doi: 10.1371/journal.pone.0005090 (PMC2662417; doi:10.1371/journal.pone.0005090)
Supplement: Text S2 — Fragility of Pxmp2-deficient peroxisomes in vitro. (0.03 MB DOC) [file pone.0005090.s009.doc]

**Supporting information**

**Note S1. Fragility of Pxmp2-deficient peroxisomes *in vitro***

In our recent research we have shown that mammalian peroxisomes are osmotically sensitive particles [1,4]. In the absence of an appropriate osmoprotectant during isolation, they suffer damage due to osmotic pressure generated by a massive flow of water into the organelles as a result of an imbalance in the content of proteins and concentration of solutes between peroxisomal lumen and the solution in which the particles are suspended. Apparently, this imbalance occurs during homogenization as the result of an abrupt decrease in the concentration of solutes outside the particles, which triggers an osmosis-driven water influx into peroxisomes.

The membrane channels may be actively involved in the osmotic behavior of peroxisomes, providing routes for water and solutes to move into and out of the particles, respectively (see Fig. S2I). The rate of this movement through relatively narrow channels is inversely dependent on the logarithm of the size of molecules (for solutes, their hydrated radii are considered [21]). Consequently, the rate of penetration of small molecules, like water, through the channel is high if compared with the movement of hydrated solutes that can be partially restricted by the size of the channel. In addition, as it is well known, the water molecules are able to diffuse spontaneously at relatively high rate across the phospholipids bilayer of the membrane without participation of any protein molecules.

If peroxisomal membrane contains several types of channels, they all can contribute to the transfer of water and solutes along concentration gradients generated during the tissue homogenization (Fig. S2J). What could be expected if one type of channel is missing? This may create troubles with the transfer of solutes owing to the fact that their movement through the channels is restricted and, as a result, can not be completely compensated for by increasing the rate of diffusion through other channels. In contrast, transfer of water will not be markedly limited due to accelerated spontaneous diffusion along the osmotic gradient and increasing flow through the rest of the channels. On the whole, disappearance of some channels will cause a delay in equilibration of the concentrations of solutes between the peroxisomes and the exterior, and in turn will produce additional osmotic pressure inside the particles, leading to more severe damage. To confirm this prediction we used PEG1500 which, as has been shown earlier [1] is an appropriate osmoprotectant for mammalian peroxisomes, aiming at preventing an osmotic lyses of the particles during homogenization of liver tissue from Pxmp2-deficient mice (see Fig. S2F-H). The results show that addition of PEG1500 to the isolation medium completely abolished the effect of Pxmp2 deficiency on the fragility of peroxisomes. This indicates a crucial role for the osmotic component in the mechanism responsible for damage of the particles caused by deletion of Pxmp2 protein.

In the living cells were peroxisomal transmembrane gradients of solutes are relatively flat, the dysfunction of some channels, e.g., Pxmp2 protein, can apparently be compensated by redundancy of pore-forming proteins in the peroxisomal membrane which may explain a subtle phenotype of Pxmp2-deficient mice. On the other hand, during homogenization the abrupt gradients of solutes are created across the peroxisomal membrane which, in the absence of appropriate osmoprotectants, leads to highly intensive overflow of water into the particles. At these conditions the dysfunction of one type of the pore-forming proteins will inevitably produce an additional osmotic damage to peroxisomes due to decrease in efficiency of the transfer of solutes across the membrane (Fig. S2I,J). The predictions described above led us to consider the high fragility of Pxmp2^-/-^ peroxisomes *in vitro* as an indication that the Pxmp2 protein is a peroxisomal membrane channel and triggered further experiments to confirm this assumption.
