# Supplementary material for: Pxmp2 Is a Channel-Forming Protein in Mammalian Peroxisomal Membrane
Source: PLoS One. 2009 Apr 7;4(4):e5090. doi: 10.1371/journal.pone.0005090 (PMC2662417; doi:10.1371/journal.pone.0005090)
Supplement: Text S3 — Latency of peroxisomal enzymes. (0.03 MB DOC) [file pone.0005090.s010.doc]

**Note S2. Latency of peroxisomal enzymes**

In comparison with the latency of enzymes located in, e.g., mitochondria and lysosomes, whose membranes are impermeable to substrates, the latency of peroxisomal enzymes is a more complex phenomenon owing to the unique permeability of peroxisomes to solutes. Cofactor-dependent enzymes display a high degree of latency in peroxisomes (see Fig. S3A), and the residual ‘free’ activity is determined by leakage of these enzymes from the particles [4]. In contrast, for peroxisomal oxidases using ‘small’ solutes as substrates (urate oxidase, L-α-hydroxyacid oxidase, D-aminoacid oxidase and others) the ‘free’ and ‘total’ activities are near equal if peroxisomes have been isolated from livers of wild-type rats [4,22] or mice (see Fig. 1D and Fig. S3A). This can be explained in terms of a high permeability rate for ‘small’ solutes across peroxisomal membrane. The latency of peroxisomal catalase, which utilizes H_2_O_2_ i.e., a ‘small’ solute, is a well known exception to the common rule. This unusual property of catalase has been attributed to the very high concentration of this extremely active enzyme in peroxisomes [22,23] and its near first order kinetics which is due to the high K_m_ for H_2_O_2_ (>1.0 M). Therefore, it is not possible in practice to reach saturation of the enzyme by increasing substrate concentration and thereby decrease the latency of catalase. In contrast, gradual inhibition of the intraperoxisomal catalase *in vitro* has led to a decline in the latency of the enzyme [23]. Theoretical assessments pointed to the role of the peroxisomal membrane as a barrier that limits the flow of H_2_O_2_ into the particles [23].

Our results indicate that Pxmp2-deficient membrane, at least *in vitro*, is a rate-limiting factor not only for catalase activity, but also for activities of cofactor-independent oxidases such as urate oxidase (Fig. 1D) and L-α-hydroxyacid oxidase (Fig. S3A). Latencies of these enzymes, like the latency of catalase, can be considered in terms of two basic parameters: velocity of permeation of the corresponding substrate across the membrane (V_1_) and velocity of conversion of this substrate into the product by the enzyme (V_2_, see Fig. S3B). Given this, the enzyme shows latency if V_2_>V_1_. This suggestion has been tested by means of oxamic acid, a specific inhibitor of urate oxidase. Treatment of Pxmp2-deficient peroxisomes with oxamic acid led to a decrease in activity of urate oxidase accompanied by a decline in the latency of the enzyme (Antonenkov V.D., unpublished observation).

One can predict that for urate oxidase, which barely leaks out of peroxisomes due to localization in nucleoids, nearly the entire free activity is determined by the velocity of substrate diffusion across the membrane. This feature of urate oxidase allows a simple approach to obtaining an approximate estimation of the steady-state concentrations of uric acid inside peroxisomes *in vitro*. In contrast to catalase, the peroxisomal oxidases follow Michaelis-Menten kinetics (Fig. S3C,D). Therefore, latency of the oxidases can be seen only if enzymes are unsaturated by substrates. At standard assay conditions for detection of the activity of urate oxidase, the concentration of urate in the incubation medium is 150 μM (marked as arrow 1 on Fig. S3C). At this substrate concentration the ‘total’ enzyme activity is close to the maximal velocity (V_max_) of the enzyme. In peroxisomes isolated from normal mouse liver, urate oxidase has consistently showed a moderate latency that comprises 10-15% of the ‘total’ activity. The residual ‘free’ activity can be used as an indicator of the steady-state concentration of uric acid inside peroxisomes (arrow 2 on Fig. S3C). This concentration is approximately two times lower than outside the particles. Deletion of Pxmp2 leads to a further decrease in the steady-state concentration of urate to a level that is more than three times lower than in the control preparation, i.e., wild-type mice (compare positions of arrows 2 and 3). As a whole, the results indicate that Pxmp2 deletion substantially affects the velocity of uric acid permeation across peroxisomal membrane although it does not entirely prevent diffusion of this compound.
